# Lipids containing medium-chain fatty acids are specific to post-whole genome duplication Saccharomycotina yeasts

**DOI:** 10.1186/s12862-015-0369-2

**Published:** 2015-05-28

**Authors:** Marine Froissard, Michel Canonge, Marie Pouteaux, Bernard Cintrat, Sabrina Mohand-Oumoussa, Stéphane E. Guillouet, Thierry Chardot, Noémie Jacques, Serge Casaregola

**Affiliations:** INRA, UMR 1318, Institut Jean-Pierre Bourgin, Saclay Plant Sciences, 78026 Versailles cedex, France; AgroParisTech, UMR 1318, Institut Jean-Pierre Bourgin, Saclay Plant Sciences, 78026 Versailles cedex, France; INRA, UMR 1319, Institut MICALIS, CIRM-Levures, 78850 Thiverval-Grignon, France; AgroParisTech, UMR 1319, Institut MICALIS, CIRM-Levures, 78850 Thiverval-Grignon, France; Université de Toulouse, INSA, UPS, INP, LISBP, 135 Av. de Rangueil, 31077 Toulouse, France

**Keywords:** Lipid, Medium-chain fatty acids, Saccharomycotina, Yeasts, Phylogeny

## Abstract

**Background:**

Yeasts belonging to the subphylum Saccharomycotina have been used for centuries in food processing and, more recently, biotechnology. Over the past few decades, these yeasts have also been studied in the interest of their potential to produce oil to replace fossil resources. Developing yeasts for massive oil production requires increasing yield and modifying the profiles of the fatty acids contained in the oil to satisfy specific technical requirements. For example, derivatives of medium-chain fatty acids (MCFAs, containing 6–14 carbons) are used for the production of biodiesels, cleaning products, lubricants and cosmetics. Few studies are available in the literature on the production of MCFAs in yeasts.

**Results:**

We analyzed the MCFA content in *Saccharomyces cerevisiae* grown in various conditions. The results revealed that MCFAs preferentially accumulated when cells were grown on synthetic media with a high C/N ratio at low temperature (23 °C). Upon screening deletion mutant strains for genes encoding lipid droplet-associated proteins, we found two genes, *LOA1* and *TGL3*, involved in MCFA homeostasis. A phylogenetic analysis on 16 Saccharomycotina species showed that fatty acid profiles differed drastically among yeasts. Interestingly, MCFAs are only present in post-whole genome duplication yeast species.

**Conclusions:**

In this study, we produced original data on fatty acid diversity in yeasts. We demonstrated that yeasts are amenable to genetic and metabolic engineering to increase their MCFA production. Furthermore, we revealed that yeast lipid biodiversity has not been fully explored, but that yeasts likely harbor as-yet-undiscovered strains or enzymes that can contribute to the production of high-value fatty acids for green chemistry.

**Electronic supplementary material:**

The online version of this article (doi:10.1186/s12862-015-0369-2) contains supplementary material, which is available to authorized users.

## Background

Depletion of fossil resources, as well as the negative environmental impact of fuel production and use, have led to a search for technologies that can generate renewable and environmentally safe alternatives. Therefore, the development of biomass-derived oil produced for energy and green chemistry purposes has become increasingly important. Biomass oil and its biodegradable by-products hold great promise for the replacement of products of fossil origin [1]. They are increasingly found in ordinary consumer goods or industrial products [2]. For example, fatty acids of high interest for green chemistry include medium-chain fatty acids (MCFAs), i.e. fatty acids with 6 to 14 carbons. MCFAs, due to their lathering and low-viscosity properties, are widely used in detergents and lubricants [3]. After esterification with alcohol, MCFAs can be used as emulsifiers for food and cosmetics. MCFA triacylglycerols are employed as solvents for flavors, surface treatment of food products and as readily digestible fat in high-energy diets. MCFAs are also valuable precursors for biodiesel because fatty acid methyl esters (FAMEs) with medium-chain length improve fuel quality [4, 5].

The major source of MCFAs is coconut or palm kernel oil [3]. MCFA production has been investigated using reverse-engineering approaches in oleaginous plants and heterologous expression of thioesterases in plants [1, 6]. In contrast, there are few reports in the literature on MCFA production by microorganisms. However, recently there has been renewed interest in this field due to the search for new tools to produce bio-based chemicals and an increasing interest in the antimicrobial activity of MCFAs. Wild-type and mutant algae have been described as good cell systems for C10:0 to C16:0 fatty acid synthesis [7]. Recent efforts have also been devoted to produce MCFAs using bacterial genetic and metabolic engineering [8, 9]. Likewise, *Yarrowia lipolytica*, a yeast that has extracellular lipase activity, is also used in the hydrolysis of coconut fat for enrichment in C8:0 to C12:0 fatty acids (FAs) [10]. MCFA biosynthesis and content in yeasts have recently been carefully documented for species belonging to the genus *Saccharomyces* that ferment sugars into alcohol. These fatty acids are intermediates in the biosynthetic pathways of volatile aromas (MCFA ethyl esters). Low-temperature fermentation (10–15 °C) and anaerobic conditions have been selected for white and rosé wine production to better develop taste and aroma. Under these conditions, cells show increased synthesis of MCFAs and triacylglycerols (TAGs) [11, 12]. These studies and more recent lipidome analyses on *S. cerevisiae* grown at various temperatures have shown that the MCFA content is very low (less than 10 *%*) and varies among *Saccharomyces* species [13, 14]. MCFAs are typically mentioned as minor fatty acids and are not detected or not shown on lipid profiles and factors controlling the homeostasis of MCFA in yeasts are currently poorly documented [15, 16]. Although improving MCFA production using metabolic and genetic engineering in *S. cerevisiae* is an emerging research topic [17], the development of yeasts for optimal MCFA production requires identifying the underlying metabolic pathways.

Yeasts are involved in many areas of biotechnology [18]. Modern taxonomy combined with comparative genomics have identified more than 1,000 yeast species [19, 20], although less than 100 species are used for biotechnology purposes [18]. Saccharomycotina yeasts form a discrete monophyletic group in the phylum Ascomycota [21]. Despite their monophyletic character, these yeasts are very diverse from a phenotypic and morphological point of view, having evolved over the last 250–900 million years [22]. Unique among the Saccharomycotina, the Saccharomycetaceae share an ancestor that underwent duplication of its genome, the so-called whole-genome duplication (WGD), followed rapidly by a massive loss of genes [23], leading to extant species with higher numbers of chromosomes than other Saccharomycotina yeast species, albeit with similar gene content. Saccharomycotina oleaginous yeasts have been studied extensively with regard to massive oil production for biofuels or chemical feedstock [24–27]. Recent studies indicate that oil content can also be significantly increased in non-oleaginous yeasts such as *S. cerevisiae* [28–30]. *S. cerevisiae* thus appears to be an attractive and promising platform for microbial oil production for industrial applications [31]. Other Saccharomycotina yeasts may also have similar potential, but they remain to be identified and characterized.

In this study, we explored the MCFA content and production in Saccharomycotina yeasts. First, we describe the MCFA content in *S. cerevisiae* BY 4741 grown in various conditions and discovered two genes, *LOA1* and *TGL3*, involved in MCFA homeostasis in cells. Second, we compare 16 Saccharomycotina yeast species and show that they have contrasting FA profiles that follow a phylogenetic pattern. In particular, using dedicated procedures for lipid extraction, we show for the first time that MCFAs are found only in post-WGD species.

## Methods

### Yeast strains and growth conditions

The yeast strains used in this study are from CIRM-Levures (http://www6.inra.fr/cirm/Levures) and Euroscarf (http://web.uni-frankfurt.de/fb15/mikro/euroscarf/) (see Table 1). Cells were routinely grown in complete medium (YP) containing 1 % (w/v) yeast extract, 2 % (w/v) peptone and 2 % (w/v) glucose. They were also grown in synthetic medium containing 0.67 % (w/v) yeast nitrogen base without amino acids and ammonium sulfate (YNB), with high nitrogen content (high-N YNB) supplemented with 5 g.L^−1^ ammonium sulfate and 0.2 % (w/v) casamino acids, 20 mg.L^−1^ uracil and 2 % (w/v) glucose, or with low nitrogen content (low-N YNB) supplemented with 0.5 g.L^−1^ ammonium sulfate, 0.2 % (w/v) casamino acids, 20 mg.L^−1^ uracil and 4 % (w/v) glucose.. All cells (except for the time course study) were grown up to the stationary phase for 48 h at 23 °C or 24 h at 28 °C at an agitation rate of 200 rpm. For the time-course study on *S. cerevisiae* and *S. uvarum*, sampled time points were selected to ensure that cells were in the early exponential phase, late exponential phase or early stationary phase, according to growth curves (see Additional file 1: Figure S1C and D).

**Table 1:** Strains used in this study

Species	CLIB number	Other name	Genotype	Origin
*Saccharomyces cerevisiae*		BY 4741 (WT)	*MATa, his3Δ, leu2Δ, met15 Δ, ura3Δ*	Euroscarf
*Saccharomyces cerevisiae*		BY 4741 *tgl3*Δ	*YMR313C::natNT2, MATa, his3Δ, leu2Δ, met15Δ, ura3Δ*	This study
*Saccharomyces cerevisiae*		BY 4741 *loa1*Δ	*YPR139C::natNT2, MATa, his3Δ, leu2Δ, met15Δ, ura3Δ*	This study
*Saccharomyces cerevisiae*	CLIB 338	S288c	*MATalpha, mal, gal2, CUP1*	CIRM-Levures
*Saccharomyces paradoxus*	CLIB 228^T^	CBS 432^T^		CIRM-Levures
*Saccharomyces uvarum*	CLIB 251^T^	CBS 395^T^		CIRM-Levures
*Saccharomyces arboricolus*	CLIB 1319^T^	CBS 10644^T^		CIRM-Levures
*Kazachstania exigua*	CLIB 179^T^	CBS 379^T^		CIRM-Levures
*Naumovozyma castellii*	CLIB 159^T^	CBS 4309^T^		CIRM-Levures
*Candida glabrata*	CLIB 298^T^	CBS 138^T^		CIRM-Levures
*Vanderwaltozyma polyspora*	CLIB 392^T^	CBS 2163^T^		CIRM-Levures
*Zygosaccharomyces rouxii*	CLIB 491^T^	CBS 732^T^		CIRM-Levures
*Kluyveromyces lactis*	CLIB 640			CIRM-Levures
*Debaryomyces hansenii*	CLIB 197^T^	CBS 767^T^		CIRM-Levures
*Millerozyma farinosa*	CLIB 492	CBS 7064		CIRM-Levures
*Pichia guilliermondii*	CLIB 734			CIRM-Levures
*Blastobotrys adeninivorans*	CLIB 1468	CBS 8244		CIRM-Levures
*Geotrichum candidum*	CLIB 918			CIRM-Levures
*Yarrowia lipolytica*	CLIB 122		*MATb, his1Δ, leu2Δ270, ura3Δ302, xpr2Δ322*	CIRM-Levures

T: type strain

### Mutant strain construction

Gene disruptions were performed by inserting heterologous DNA in genomic locations using a simple polymerase chain reaction (PCR)-based strategy as described in Janke et al. [32].

### Growth test

Cells were grown overnight in 5 mL of YP + 2 % glucose medium and were spotted on plates containing agar YP + 2 % glucose medium. Plates were incubated 24 h at 28 °C or 48 h at 23 °C. The first drop contained around 500 cells and each subsequent drop was diluted six-fold compared to the prior drop.

### Lipid extraction

Cells (corresponding to 50 mg dry weight (dw)) were collected by centrifugation, washed with water, disrupted with a One Shot Cell Disrupter (Constant System LDT) and freeze-dried for 72 h. They were then processed according to Folch et al. [33]. Briefly, 2.5 mL of chloroform:methanol (2:1, v/v) was added to the pellet and the cells were disrupted by vortexing 5 min using glass beads (0.45 mm). After 1 h incubation with shaking, the extract was centrifuged 5 min at 500 × g and the supernatant was recovered in a new tube. The extraction was repeated twice. The supernatants were pooled and mixed with 2.5 mL of 0.9 % NaCl. The organic (lower) phase was collected after centrifugation at 500 × g for 5 min and washed once with 1.5 mL of chloroform:methanol:water (3:48:47, v/v/v) [34]. The organic solvents were evaporated under stream of N_2_ and lipids were solubilized in 1 mL of chloroform:methanol (2:1, v/v) or hexane:diethyl ether:acetic acid (80:20:1, v/v/v).

### Lipid fractionation

To fractionate lipids, 500 μL of lipids solubilized in MIX1 (hexane:diethyl ether:acetic acid, 80:20:1, v/v/v) were loaded on a cyanopropylsilyl solid-phase extraction column (Grace) previously equilibrated with 10 mL of MIX1. The column was washed first with 5 mL of MIX1 to recover the nonpolar lipids and then the polar lipids were eluted using 5 mL of MIX2 (chloroform:methanol:water, 40:10:1, v/v/v). The organic solvents contained in each fraction were evaporated under stream of N_2_. Samples were then processed for gas chromatography (GC) as described below.

### Lipid analysis

Lipids (20 μL) solubilized in chloroform:methanol (2:1, v/v) were separated by thin layer chromatography (TLC) on high-performance TLC silica-coated aluminum plates (Merck) using two mobile phases successively, petroleum ether:diethyl ether:acetic acid (10:10:0.4, v/v/v) and petroleum ether:diethyl ether (49:1, v/v) until the solvent front reached about 15 cm and 1 cm from the top of the plate, respectively [35, 36]. Lipid classes were visualized using the MnCl_2_ charring method: silica plates were incubated for 1 min in a solution containing 120 mL of methanol, 120 mL of water, 0.8 g of MnCl_2_ and 8 mL of sulfuric acid and then heated in an oven at 100 °C until dark lipid spots appeared. Lipid identification was based upon migration obtained for lipid standards (mix of phospholipids, sterols, triacylglycerols and sterols esters at 4 μg.μL^−1^ each, Sigma-Aldrich). Lipid staining was recorded using the LAS-3000 imaging system and MultiGauge software from Fujifilm.

### Fatty acid quantification using gas chromatography

Fatty acid quantifications were performed on two types of samples: freeze-dried cells or dried lipids obtained after extraction and fractionation (see relevant sections above). Cells (corresponding to 20 mg dw) were collected by centrifugation, washed with water and freeze-dried for 72 h. The pellet was disrupted by vortexing in the presence of 0.45 mm glass beads and 2 mL of 2.5 % (v/v) sulfuric acid in methanol. Heptadecanoic acid (Sigma-Aldrich) was added (100 μg for each sample) as an internal standard for quantification. Alternatively, dried lipid fractions were treated with 2 mL of 2.5 % (v/v) sulfuric acid in methanol without an internal standard. All the samples were heated for 90 min at 80 °C. FAMEs were extracted by adding 3 mL of water, 1 mL of hexane, followed by vigorous shaking and centrifugation at 1000 × g for 10 min. Samples of the organic upper phase were separated using GC with a 7890A chromatograph (Agilent) with a Factor Four VF-23 ms 30 mm × 0.25 mm capillary column (Agilent). The carrier gas was helium at an inlet pressure of 1 mL⋅min^−1^. The column temperature program started at 40 °C for 1 min, ramping to 120 °C at 40 °C⋅min^−1^, holding 1 min at 120 °C, ramping to 210 °C at 3 °C⋅min^−1^ and holding 10 min at 210 °C. Identification of FAME peaks was based upon retention times obtained for standards (Sigma-Aldrich). Quantification was performed by flame ionization detection (FID) at 270 °C. The total amount of fatty acids was calculated from the ratio between the sum of FAME peak areas and the heptadecanoic acid methyl ester peak area.

### Identification of LPLAT sequences and phylogenetic analysis

Lysophospholipid acyltransferase (LPLAT) gene family members were extracted from various databases (NCBI, Génolevures) using the Blastp program or from Scannel *et al.* [37]. *S. cerevisiae* protein sequences from the Saccharomyces Genome Database (SGD) were used as bait (Additional file 5: Table S2)*. Geotrichum candidum* gene sequences were obtained from the complete genome sequence (Morel et al., in preparation). Sequence alignments were generated using MUSCLE ver. 3.7 implemented in phylogeny.fr, using default parameters [38] or MAFFT ver. 7 (http://mafft.cbrc.jp/alignment/server/), using blosom62 matrix and default parameters and were manually adjusted with GeneDoc (http://www.nrbsc.org/gfx/genedoc/). Phylogenetic trees were reconstructed with the PhyML program implemented in phylogeny.fr, using default parameters [38] or the Maximum Likelihood program [39] implemented in MEGA6 [40]. Phylogenetic trees were visualized using NJPlot [41]. Multiple Sequence Alignment raw data were available in Additional files 2 and 3.

## Results

### Effect of culture conditions on MCFA content in *S. cerevisiae*

**Fig. 1:**
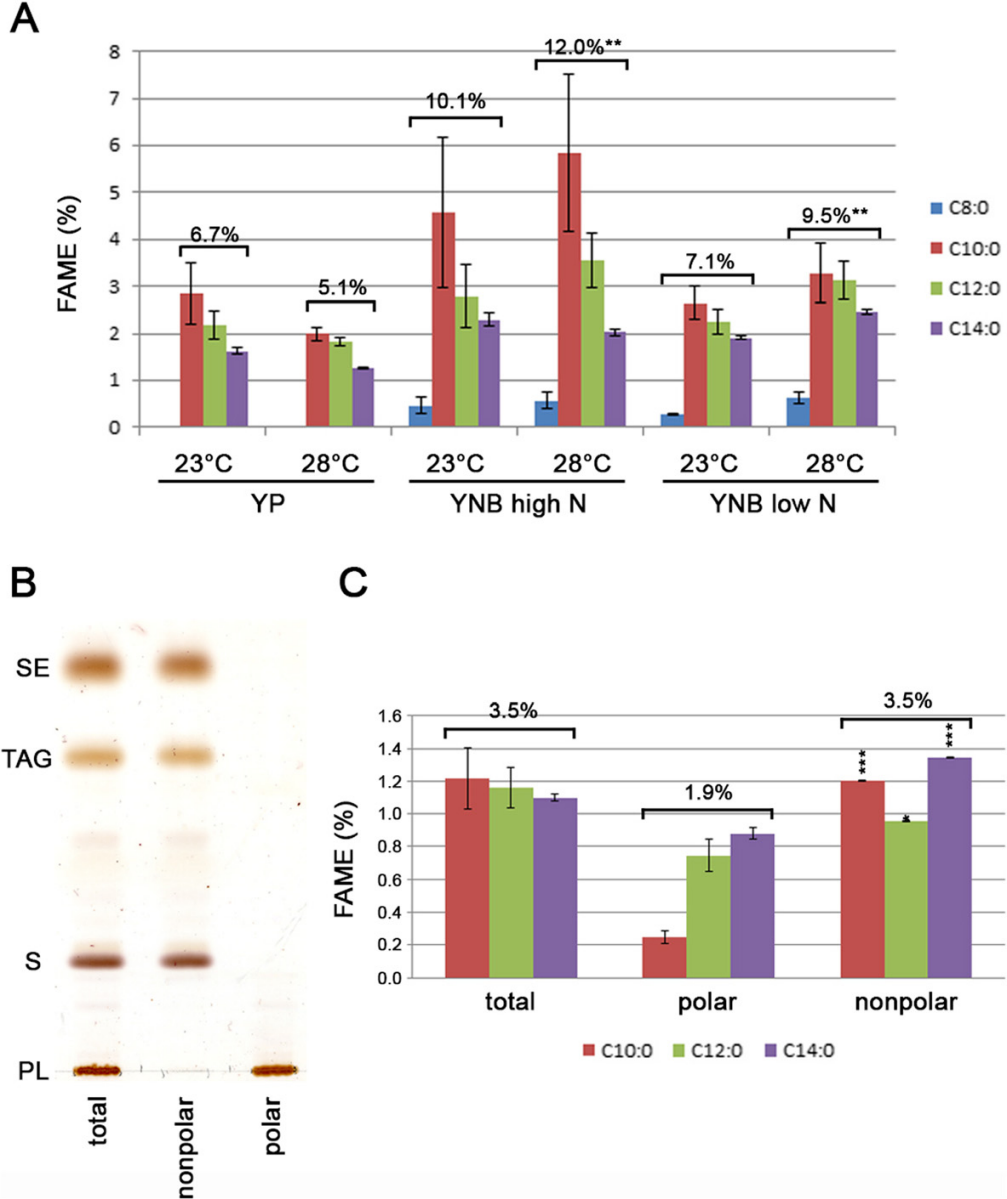
Relative MCFA content in wild-type *S. cerevisiae*. Relative amount of MCFAs (C8:0 to C14:0) in BY 4741 grown at 23 °C or 28 °C in complete medium (YP) or synthetic media (YNB) with high or low nitrogen was determined using gas chromatography (**A**). Lipids from cells grown at 23 °C in YP medium were extracted using the Folch method. Polar and nonpolar lipids were then separated using solid-phase extraction. Total lipids and polar and nonpolar fractions were analyzed using thin layer chromatography (**B**) and gas chromatography (**C**). FAME, fatty acid methyl ester; SE, sterol ester; TAG, triacylglycerol; S, sterol; PL, phospholipids. Significant difference according to Student’s *t*-test, ***P < 0.001, **P < 0.01, *P < 0.05

To obtain more information on the MCFA content in *S. cerevisiae*, we performed total fatty acid analysis on cells grown in complete (YP) or synthetic (YNB) media (high or low nitrogen) and at 23 °C for 48 h or at 28 °C for 24 h. The high-N and low-N synthetic media were designed so that the stationary phase was reached after total consumption of glucose or nitrogen, respectively. Two culture temperatures were tested because lower culture temperatures are known to increase MCFA content in yeast. We chose 23 °C as the minimal testing temperature due to the impaired growth phenotype of some strains used in the study when culture conditions are not favorable (see Additional file 1: Figure S1A and B for growth curve details). For GC analysis, FAMEs were obtained by direct transmethylation on freeze-dried samples followed by hexane extraction. We chose this protocol to maximize the recovery of MCFAs because it does not include total lipid extraction using the Folch method before transmethylation and there is no concentration of FAMEs after transmethylation. We confirmed that concentration steps using drying procedures after transmethylation lead to a total (for C8:0 FAME) or a partial loss (>50 % for C10:0, > 20 % for C12:0 FAME) of volatile medium-chain FAMEs. We measured total FA and the relative amount of MCFAs for all tested culture conditions (Additional file 4: Table S1). Temperature influenced FA content with a significant increase (+22 % for YP) at 23 °C (Table 2). For cultures grown at 28 °C, there was 16 % and 25 % increase in FA content in cells grown in high-N and low-N synthetic media, respectively, compared with cells grown in YP medium. At 23 °C, the nature of the medium did not significantly change the FA content. We did not observe any significant changes due to nitrogen content in the WT strain. Regarding MCFAs, culture conditions did not have a strong effect on the relative amount of C8:0 to C14:0 FA (Fig. 1A). C8:0 FAs were observed only in cells grown on synthetic media. Furthermore, when cultures were grown at 28 °C, the high-N and low-N synthetic media were significantly (p < 0.01) more favorable for cell MCFA content than the complete (YP) medium.

**Table 2:** Total fatty acid content in the wild-type (WT), *tgl3*Δ and *loa1*Δ strains grown in various conditions

Strain	Media	Temperature (°C)	Fatty acid (μg FAME.mg^−1^ dw)	Relative to WT	28 °C vs 23 °C	Relative to YP 28 °C	Relative to YP 23 °C	High N vs low N
WT	YP	23	54.92 ± 4.62		+22 %*			
WT	YP	28	45.05 ± 1.87					
WT	High-N YNB	23	56.13 ± 6.03		+7 %		+2 %	
WT	High-N YNB	28	52.38 ± 5.29			+16 %*		
WT	Low-N YNB	23	62.19 ± 2.16		+10 %*		+13 %	+11 %
WT	Low-N YNB	28	56.39 ± 2.73			+25 %***		+8 %
*tgl3*Δ	YP	23	69.65 ± 4.19	+27 %*	+27 %***			
*tgl3*Δ	YP	28	54.70 ± 1.22	+21 %**				
*tgl3*Δ	High-N YNB	23	71.59 ± 10.01	+27 %	+24 %		+3 %	
*tgl3*Δ	High-N YNB	28	57.71 ± 6.4	+10 %		+5 %		
*tgl3*Δ	Low-N YNB	23	77.54 ± 2.17	+25 %***	+10 %***		+11 %**	+8 %
*tgl3*Δ	Low-N YNB	28	70.13 ± 1.94	+25 %**		+28 %***		+22 %*
*loa1*Δ	YP	23	60.73 ± 1.73	+10 %	+23 %***			
*loa1*Δ	YP	28	49.21 ± 0.63	+9 %*				
*loa1*Δ	High-N YNB	23	52.81 ± 7.86	−6 %	nd		−13 %*	
*loa1*Δ	High-N YNB	28	nd			nd		
*loa1*Δ	Low-N YNB	23	70.56 ± 4.06	+13 %*	+27 %***		+16 %*	+34 %**
*loa1*Δ	Low-N YNB	28	55.44 ± 4.73	−2 %		+11 %*		nd

Data are expressed as the mean ± SE (n = 3). Significant differences according to Student’s *t*-test

***P < 0.001

**P < 0.01

*P < 0.05

### MCFAs are more abundant in neutral lipids than in phospholipids in *S. cerevisiae*

We then investigated the nature of the lipids containing MCFAs. To do so, we extracted and fractionated the lipids from cells grown at 23 °C in complete media. We obtained two fractions containing polar lipids, mainly phospholipids (PLs) and nonpolar lipids, sterols (Ss), sterol esters (SEs) and TAGs (Fig. 1B). Then, we analyzed the FA composition of each fraction using GC after transmethylation of dried lipid samples. We compared the amount of MCFAs detected using direct transmethylation with that obtained after lipid extraction. Quantification revealed fewer (p < 0.01) MCFAs when extracted using the Folch method than when detected by direct transmethylation: 3.5 % compared to 6.7 %, respectively (Figs. 1A and C). This difference may be due to a loss of MCFAs esterified on molecules other than lipids or a loss of some lipids that are difficult to extract using the chloroform:methanol procedure. These results confirm that extraction procedures greatly affect MCFA recovery as discussed above. Comparison of MCFA content in the polar and nonpolar fractions revealed that the FA composition was different in the two fractions with a higher proportion of MCFAs in nonpolar lipids (3.5 % vs. 1.9 %) corresponding to FAs from SEs and TAGs (Fig. 1C). All MCFA species (from C10:0 to C14:0 FAs) were significantly affected.

### *loa1*Δ and *tgl3*Δ, strains deficient in lipid droplet proteins over-accumulate fatty acids at low temperature and under nitrogen starvation

**Fig. 2:**
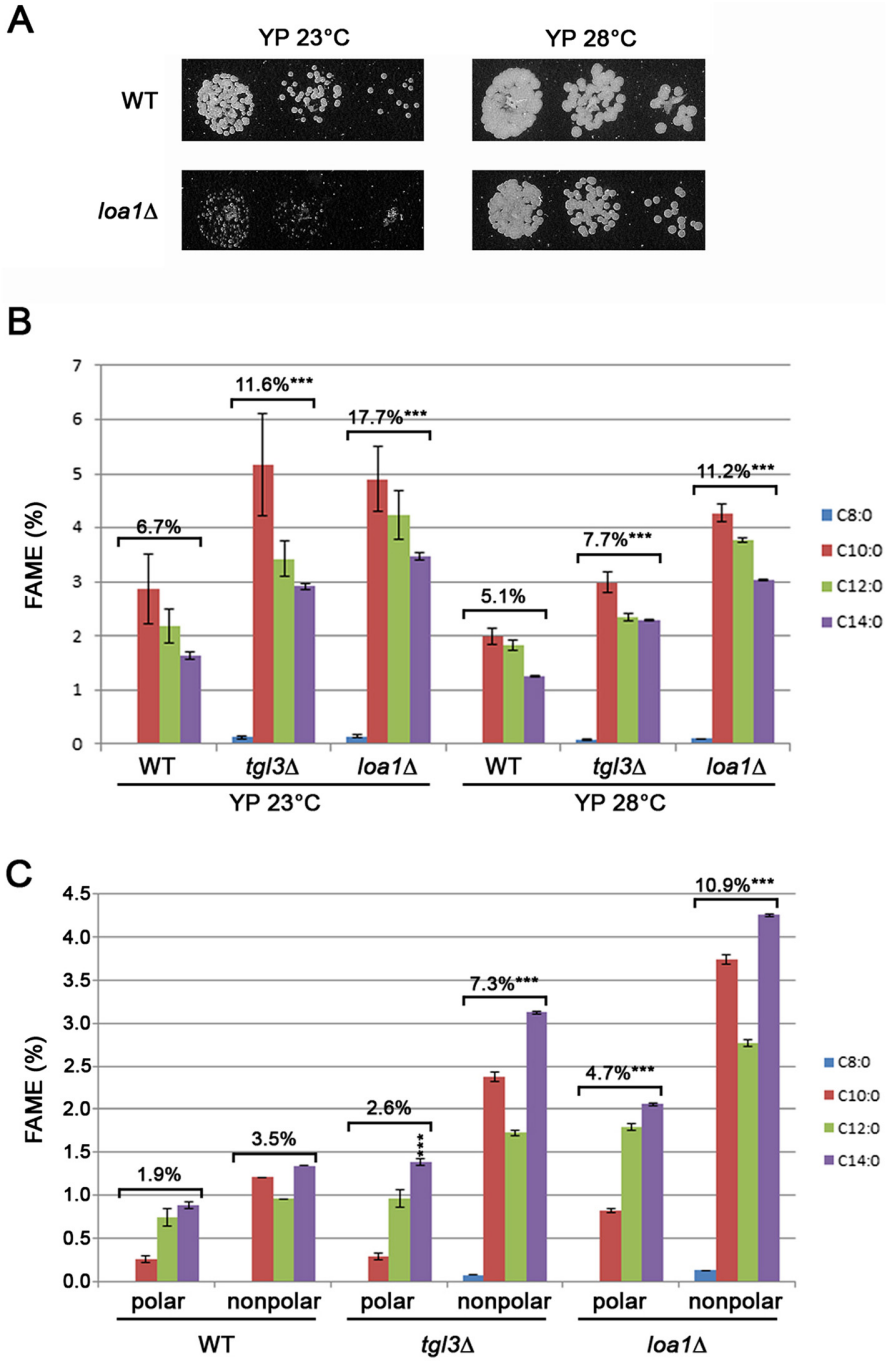
Relative MCFA content in mutant *tgl3*Δ and *loa1*Δ strains. Growth tests on solid YP medium at 23 °C and 28 °C were performed on *loa1*Δ cells (**A**). Relative amount of MCFAs (C8:0 to C14:0) in the wild-type (WT), *tgl3*Δ and *loa1*Δ grown at 23 °C or 28 °C in complete medium (YP) was determined using gas chromatography (**B**). Lipids from wild-type (WT) and mutant cells grown at 23 °C in YP medium were extracted using the Folch method. Polar and nonpolar lipids were then separated using solid-phase extraction. Total lipids and polar and nonpolar fractions were analyzed using gas chromatography (**C**). FAME, fatty acid methyl ester. Significant difference according to Student’s *t*-test, ***P < 0.001

In cells, neutral lipids (TAGs and/or SEs) are stored in organelles called lipid droplets (LDs) [42–45]. The neutral lipids are enclosed in a monolayer of phospholipids. LDS contain a number of proteins that vary considerably with species [15, 16, 46]. We investigated the role of some of these LD-associated proteins on LD dynamics and MCFA content in cells. We thus analyzed the total FA content in *S. cerevisiae* mutant cells grown in various conditions. We focused on two mutant strains, *tgl3*Δ and *loa1*Δ because Tgl3p and Loa1p are enzymes involved in lipid modification. Tgl3p is the major TAG lipase in *S. cerevisiae* [47] and has also been described as a lysophosphatidylethanolamine acyltransferase [48]. Loa1p is also an acyltransferase, but has lysophosphatidic acid acyltransferase activity [16]. GC analysis revealed that the *tgl3*Δ mutant over-accumulates FA in most of the culture conditions tested (Table 2 and Additional file 1: Figure S1 for growth curves). Significant differences (around a 25 % increase in total FAs) compared to the WT strain were observed for cells grown in YP and in low-N YNB at 23 °C and 28 °C. Low temperature and nitrogen deficiency positively influenced total FA content. We performed the same analysis on the *loa1*Δ mutant, but there was a growth defect for this strain on all media, as illustrated in Fig. 2A. This growth defect did not provide sufficient biological material for GC analysis for *loa1*Δ mutants grown in liquid high-N YNB medium at 28 °C. For cultures grown in high-N YNB medium at 23 °C, FA analysis revealed that this medium was not favorable for lipid accumulation, because there was a decrease in FA content compared with cultures performed in YP (−13 %) or low-N YNB (−34 %) at 23 °C. Nevertheless, total FA content increased (around +10 %) in the *loa1*Δ mutant grown in YP at 28 °C and in low-N YNB at 23 °C (Table 2). In the *loa1*Δ mutant, as for the *tgl3*Δ mutant, low temperature was more favorable for FA accumulation (+23 % in YP and + 27 % in low-N YNB) than high temperature.

### Neutral lipid MCFA content is higher in *loa1*Δ and *tgl3*Δ mutants

We then investigated the MCFA content of the mutant strains. For the *tgl3*Δ and *loa1*Δ strains, there was a significant increase in MCFA content and we detected some C8:0 FAs in cells grown in YP medium (Fig. 2B), with a maximum of 0.15 % for *loa1*Δ grown at 23 °C. At 23 °C, the MCFA content was 6.7 %, 11.6 % and 12.6 % in the WT, the *tgl3*Δ mutant and the *loa1*Δ mutant, respectively. At 28 °C, MCFA content was 5.1 %, 7.7 % and 11.2 % in the WT, the *tgl3*Δ mutant and the *loa1*Δ mutant, respectively. There were also significant differences in the *tgl3*Δ mutant grown in high-N YNB medium at 28 °C and for the *loa1*Δ mutant grown in low-N YNB at 23 °C compared to the WT (data not shown). Analysis of polar and nonpolar lipids extracted from cells grown in YP at 23 °C revealed that the relative amount of MCFAs increased in both lipid fractions in the mutants. There was a greater increase in the relative amount of MCFAs in the nonpolar lipid fraction than in the polar fraction, with a 2-fold and 3.5-fold increase in *tgl3*Δ and *loa1*Δ cells, respectively (Fig. 2C).

### Lipid-containing MCFAs are only present in post-WGD yeasts

**Fig. 3:**
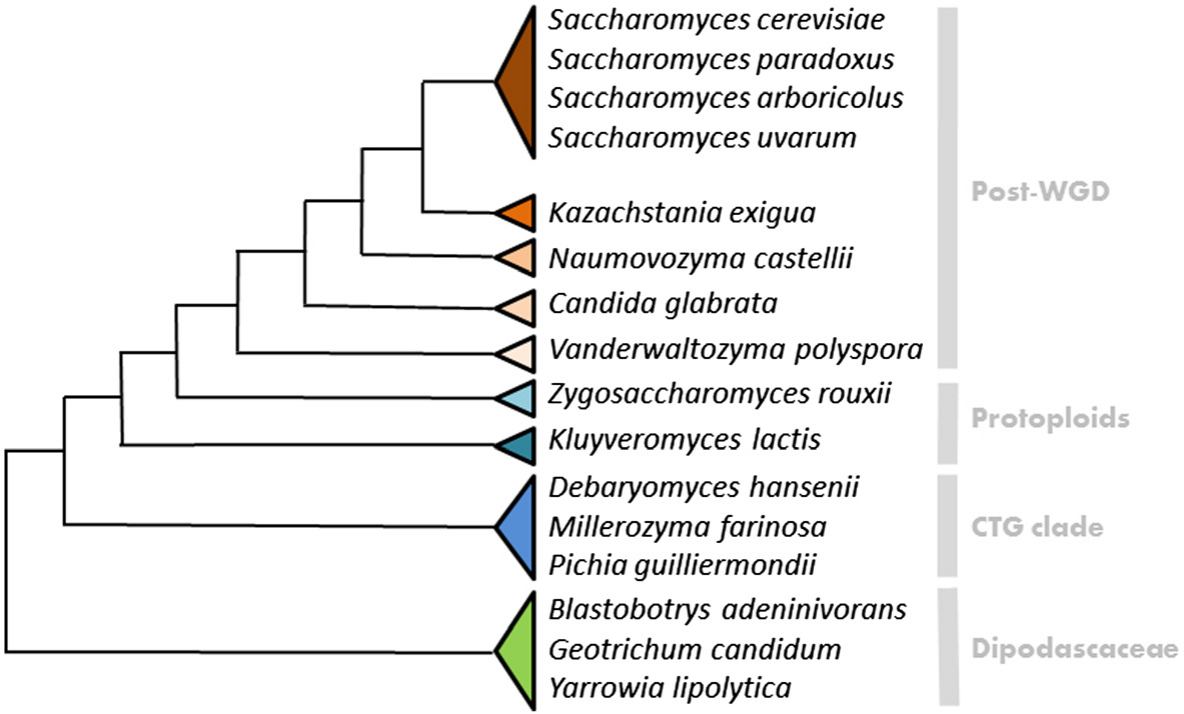
Relative phylogenetic positions of the analyzed species. The schematized phylogenetic tree indicates the evolutionary relationships of the species analyzed in this study. It was derived from Dujon [64] and additional studies (Serge Casaregola, unpublished). The clades to which species belong are indicated

**Fig. 4:**
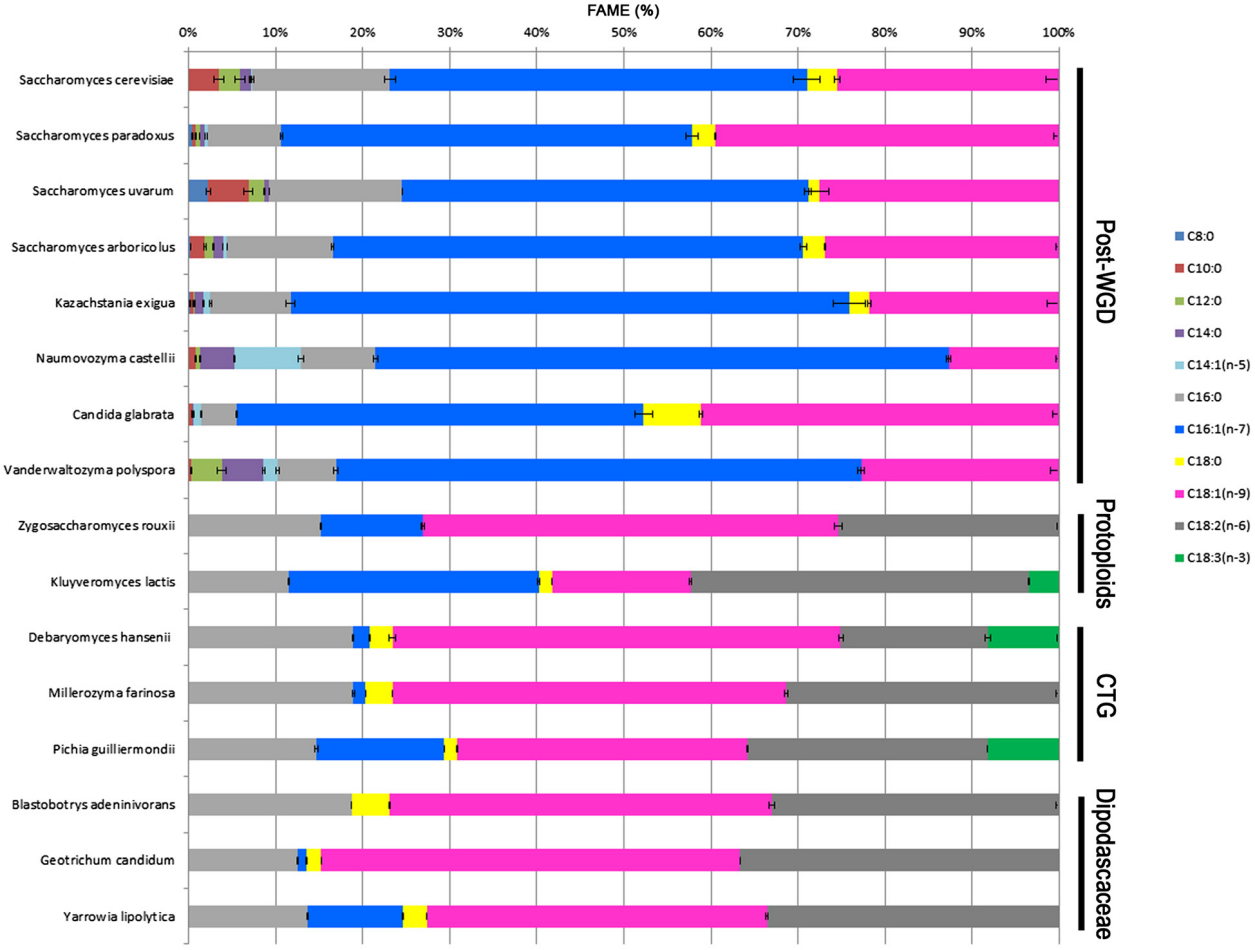
Relative amount of total fatty acids in 16 Saccharomycotina yeasts. Cells were grown at 28 °C in complete medium (YP). Complete fatty acid profiles were determined using gas chromatography after direct transmethylation on freeze-dried cells

**Fig. 5:**
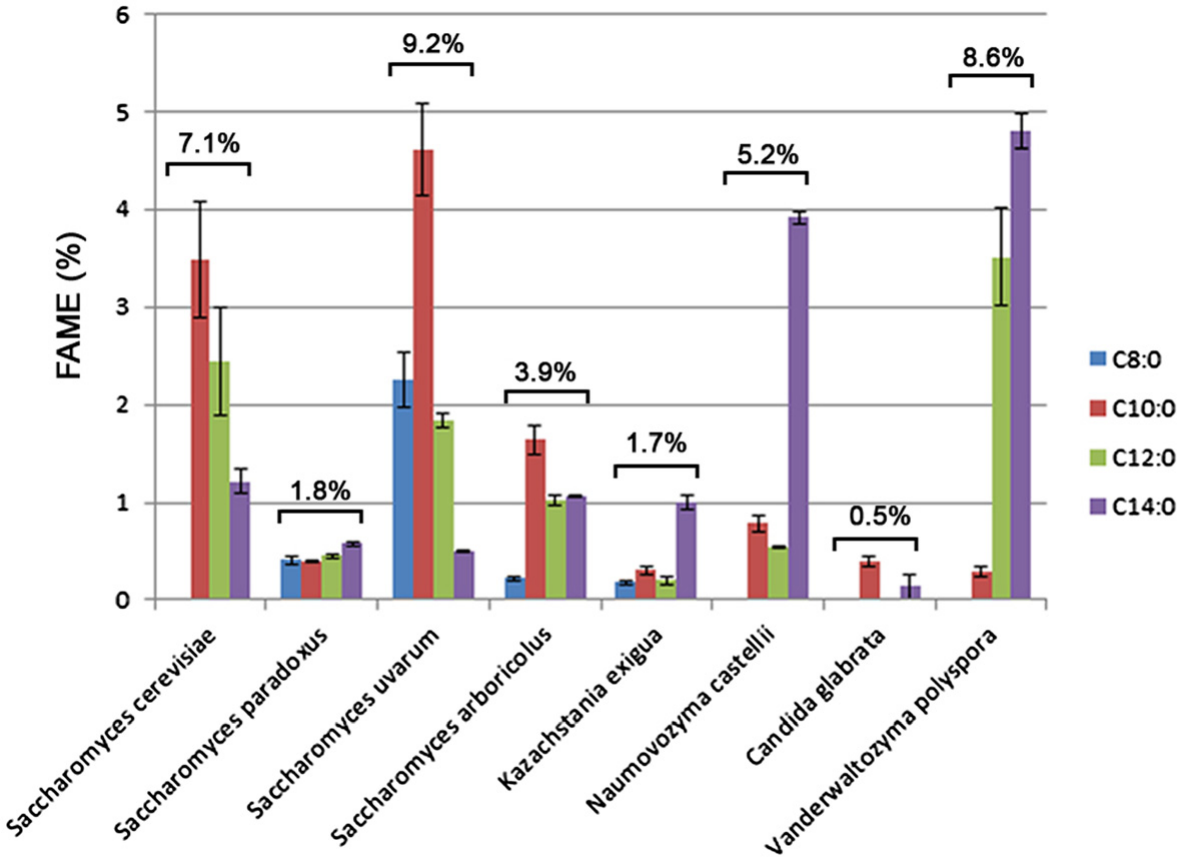
Relative MCFA content in Saccharomycotina yeasts. Relative amount of MCFAs (C8:0 to C14:0) in cells grown at 28 °C in complete medium (YP) was determined using gas chromatography

**Fig. 6:**
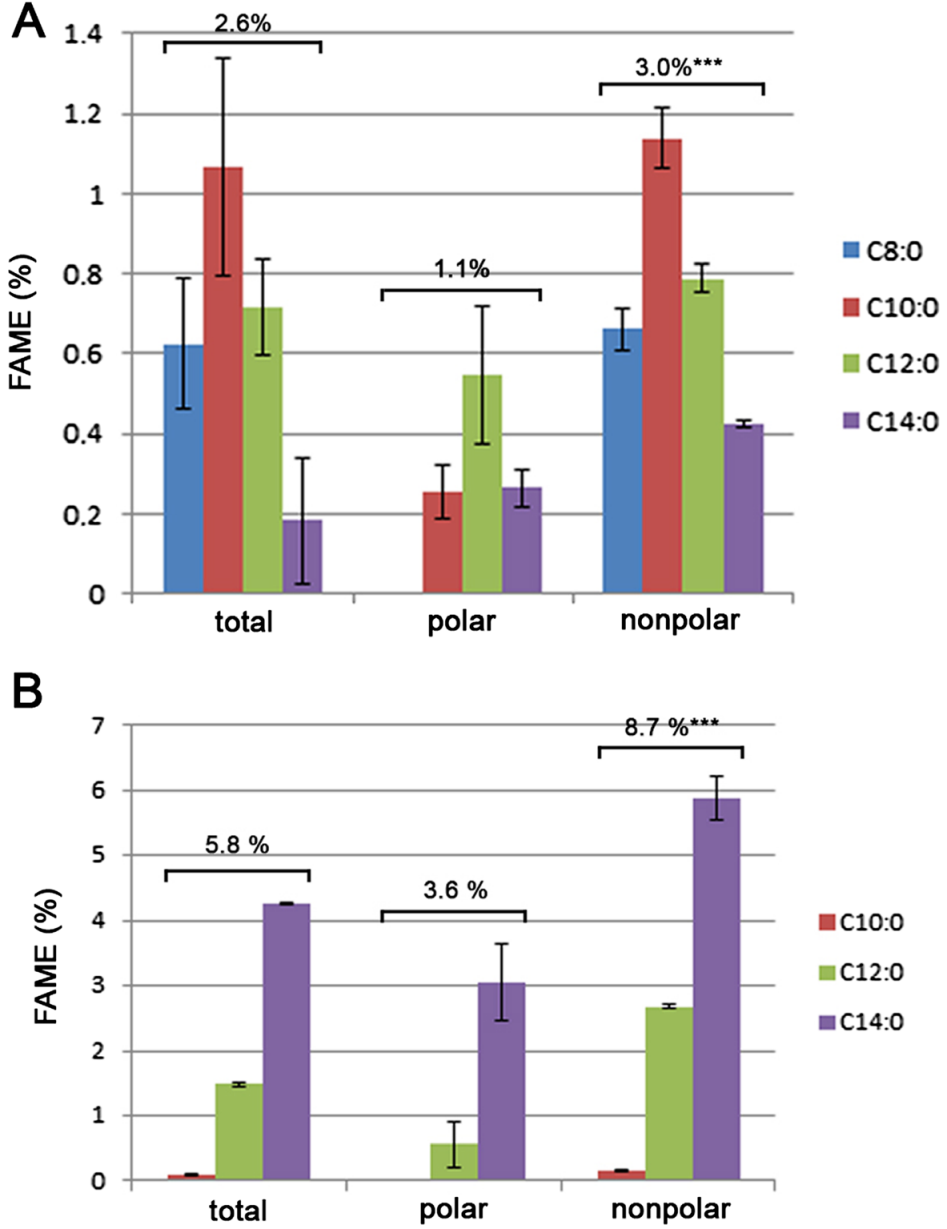
Relative MCFA content in polar and nonpolar lipids extracted from *Saccharomyces uvarum* and *Vanderwaltozyma polyspora.* Lipids from *S. uvarum* (**A**) and *V. polyspora* (**B**) cells grown at 28 °C in complete medium were extracted using Folch method. Polar and nonpolar lipids were then separated using solid-phase extraction. Total lipids and polar and nonpolar fractions were analyzed using gas chromatography. FAME, fatty acid methyl ester. Significant differences between polar and nonpolar fractions according to Student’s *t*-test, ***P < 0.001

Given the interest in single cell oil, numerous yeasts have been studied for their lipid content and their capacity to grow on lipid substrates. FA profiles have been published for some of these yeasts of biotechnological interest such as *Y. lipolytica* [49], *Komagataella pastoris* (also cited as *Pichia pastoris*) [50] or *Kluyveromyces lactis* [51]. In these species, FA profiles varied considerably with variable amounts of PUFA, C18:2 for *Y. lipolytica*, C18:2 and C18:3 for *K. pastoris* or *K. lactis*. However, no MCFAs have been described in these yeasts. To explore the FA content/production diversity of yeasts in detail, we obtained the FA profile for 16 species spanning the Saccharomycotina subphylum. The analysis included post-WGD species, protoploid species and species belonging to the so-called CTG clade and the Dipodascaceae family (Fig. 3). Cultures were performed in YP at 28 °C for 24 h because these conditions were favorable (equivalent growth curves) for all the species considered and gave us sufficient biological material for subsequent analyses. Quantification revealed contrasting FA contents even within a given clade, such as the protoploid species (Table 3). The lowest FA content was found in *Debaryomyces hansenii* (34.10 ± 0.47 μg FAME.mg^−1^ dw) and the highest FA content was found in *Saccharomyces paradoxus* (93.21 ± 2.14 μg FAME.mg^−1^ dw) (Table 3). We also considered the FA profiles in these strains and the proportion of MCFAs and PUFAs varied dramatically according to clade (Fig. 4). All the post-WGD strains contained MCFAs and were devoid of PUFAs. In contrast, the other Saccharomycotina species had PUFAs, but no MCFAs. The three Dipodascaceae species analyzed did not have C18:3 FAs. Profiles were more complex for the CTG and protoploid yeasts. In the same clade, we observed strains containing either C18:2 and C18:3 or only C18:2. We considered in detail the amount of MCFAs in these strains and observed high variability, between 0.5 % total FA in *Candida glabrata* and 9.2 % in *Saccharomyces uvarum*. The proportion of each FA species varied among the yeast species with a high C8:0 + C10:0 content in *S. uvarum* and a high C12:0 + C14:0 content in *Vanderwaltozyma polyspora* (Fig. 5). We compared the MCFA content in the polar and nonpolar fractions for *S. uvarum* and *V. polyspora*. The analysis revealed different FA compositions in the two fractions with a significantly (p < 0.001) higher proportion of MCFAs in nonpolar lipids for these two species. In particular, C8:0 FAs were present in nonpolar lipids and not in polar lipids for *S. uvarum* (Fig. 6A). Similarly, C10:0 FAs were only observable in nonpolar lipids in *V. polyspora* (Fig. 6B).

**Table 3:** Total fatty acid content in the tested strains

Species	Phylogenetic position	Fatty acid content (μg FAME.mg^−1^ d.w.)
*Saccharomyces cerevisiae*	Post-WGD	58.27 ± 5.53
*Saccharomyces paradoxus*	Post-WGD	93.21 ± 2.14
*Saccharomyces uvarum*	Post-WGD	45.53 ± 1.10
*Saccharomyces arboricolus*	Post-WGD	74.45 ± 1.91
*Kazachstania exigua*	Post-WGD	63.53 ± 5.92
*Naumovozyma castellii*	Post-WGD	62.64 ± 2.76
*Candida glabrata*	Post-WGD	46.41 ± 1.37
*Vanderwaltozyma polyspora*	Post-WGD	74.91 ± 3.82
*Zygosaccharomyces rouxii*	Protoploid	37.26 ± 0.37
*Kluyveromyces lactis*	Protoploid	68.04 ± 1.02
*Debaryomyces hansenii*	CTG	34.10 ± 0.47
*Millerozyma farinosa*	CTG	63.17 ± 2.04
*Pichia guilliermondii*	CTG	45.35 ± 2.22
*Blastobotrys adeninivorans*	Dipodascaceae	50.12 ± 0.43
*Geotrichum candidum*	Dipodascaceae	46.80 ± 0.96
*Yarrowia lipolytica*	Dipodascaceae	39.16 ± 0.96

FAME, fatty acid methyl ester; WGD, whole genome duplication

**Fig. 7:**
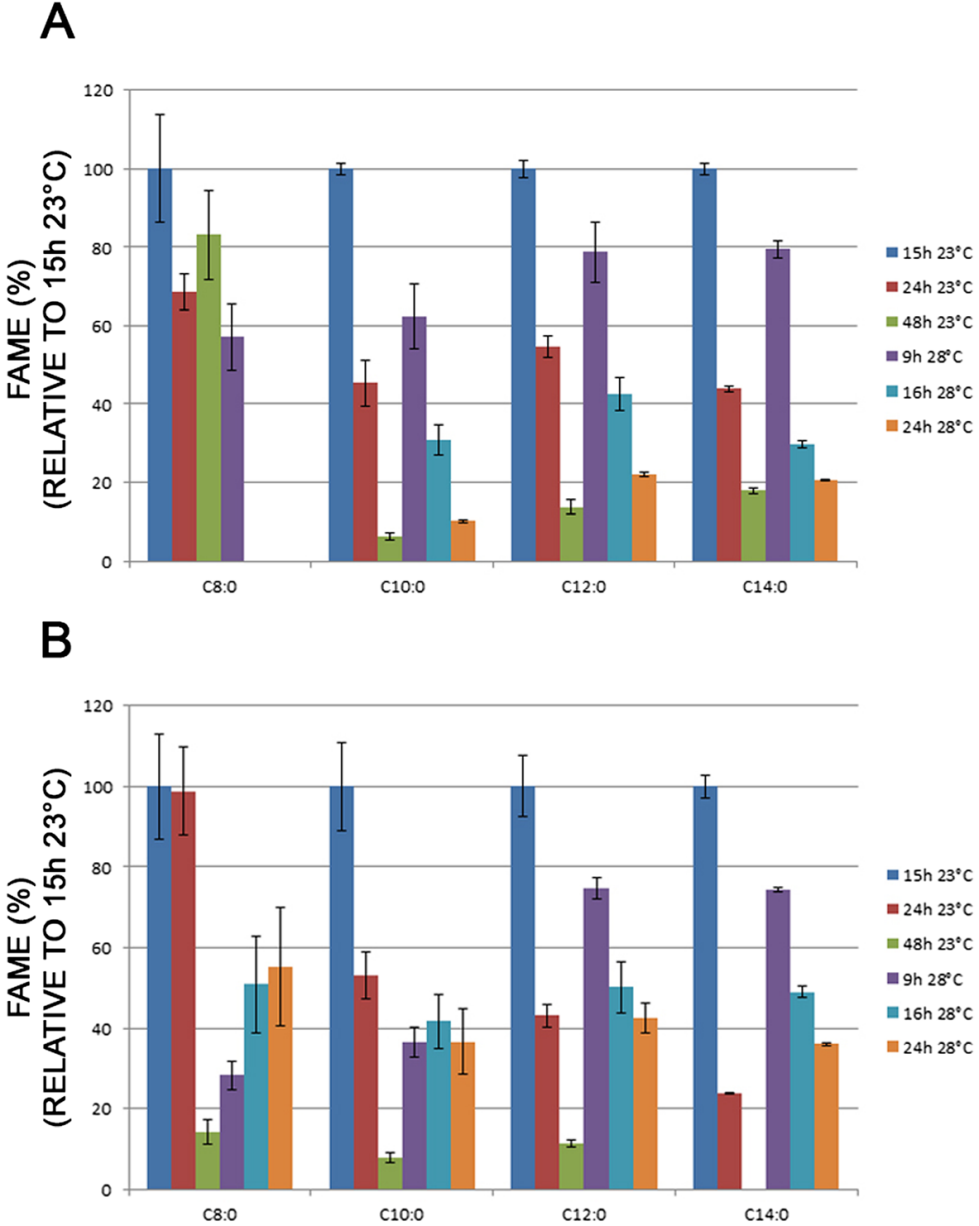
Time course study of relative MCFA content in *Saccharomyces cerevisiae* and *Saccharomyces uvarum* grown in YP medium at 23 °C and 28 °C. Relative amount of MCFAs (C8:0 to C14:0) in *S. cerevisiae* (**A**) and *S. uvarum* (**B**) grown 15 h, 24 h and 48 h at 23 °C or 9 h, 16 h and 24 h at 28 °C in complete medium (YP) was determined using gas chromatography. FAME, fatty acid methyl ester

Then, we investigated MCFA content with regard to culture conditions. We selected *S. cerevisiae* and *S. uvarum* grown in YP medium at 23 °C and 28 °C for a time-course study. Cells were harvested at three time points corresponding to the mid-exponential phase, the late exponential phase and the early stationary phase (See Additional file 1: Figure S1 for growth curves). The FA analysis revealed that the MCFA content was sensitive to temperature and growth phase with a maximum content for cells grown at 23 °C and sampled at the early exponential phase (Fig. 7). For *S. cerevisiae* grown at 28 °C, C8:0 FAs were only produced by cells during the early exponential phase (Fig. 7A). There was no decrease in C8:0 and C10:0 FA during growth in *S. uvarum* grown at 28 °C (Fig. 7B).

### Comparative genomics of LPLATs in Saccharomycotina

Given the high variability in lipid profiles among species and the impact of the deletion of the acyltransferase gene *LOA1* on MCFA content in *S. cerevisiae*, we investigated the Saccharomycotina phylogeny of the lysophospholipid acylytransferase (LPLATs) genes, such as *LOA1*, which are key enzymes in lipid metabolism. We therefore used a comparative genomics approach; known *S. cerevisiae* LPLAT genes *SCT1* (*YBL011W*)*, GPT2* (*YKR067W*)*, CST26* (*YBR042C*)*, YDR018C, SLC1* (*YDL052C*)*, DGA1* (*YOR245C*)*, TAZ1* (*YPR140W*)*, LOA1* (*YPR139C*) and *MUM3* (*YOR298W)* were used as bait in Blastp searches to retrieve orthologous sequences from available genomes. We considered a total of 26 species to include most of the major Saccharomycotina lineages. The accession numbers of the selected sequences are listed in Additional file 5: Table S2. Phylogenetic analysis of the LPLAT family revealed high conservation of these genes with the presence of orthologous genes in most of the species considered (Additional file 6: Figure S2; Additional file 7: Figure S3; Additional file 8: Figure S4; Additional file 9: Figure S5; Additional file 10: Figure S6; Additional file 11: Figure S7; Additional file 12: Figure S8, Additional file 3). We observed a variable number of orthologs in the species studied according to the gene considered (Additional file 5: Table S2). As expected for *Candida albicans*, two orthologs were found given that all the isolates of this species are naturally diploid heterozygotes. Likewise, in *Millerozyma farinosa*, two orthologs for each screened *S. cerevisiae* gene were always found due to the hybrid nature of its genome [52]. However, the *GPT2* orthologs were missing in this strain (see below). We observed a few cases of duplicated genes such as *CST26* in *Naumovozyma castelli* and *Vanderwaltozyma polyspora* as well as *SLC1* in *V. polyspora*. These duplications are very likely the remains of the WGD known to have been followed by independent gene loss [53]. For the other species, most of the genes analyzed were single-copy genes. Some species have also lost genes; two cases were observed: *S. arboricolus* with four genes missing out of the nine genes tested and *Ogatea polymorpha* with two genes missing. More specifically, *M. farinosa* strain CLIB 492 did not carry any *GPT2* orthologs. The presence of two *SCT1* genes in this hybrid strain may compensate for the loss of *GTP2*. On the other hand, *S. arboricolus* carried a *GPT2* gene, but no *SCT1* gene. Interestingly, YDR018C was only present in *Saccharomyces*, except *S. arboricolus*, suggesting that this gene was conserved only in *Saccharomyces* during evolution, while the ortholog was lost in all the other species tested. Finally, *GPT2* and *MUM3* were not present in any species of the family Dipodascaceae or in basal fungal species.

**Fig. 8:**
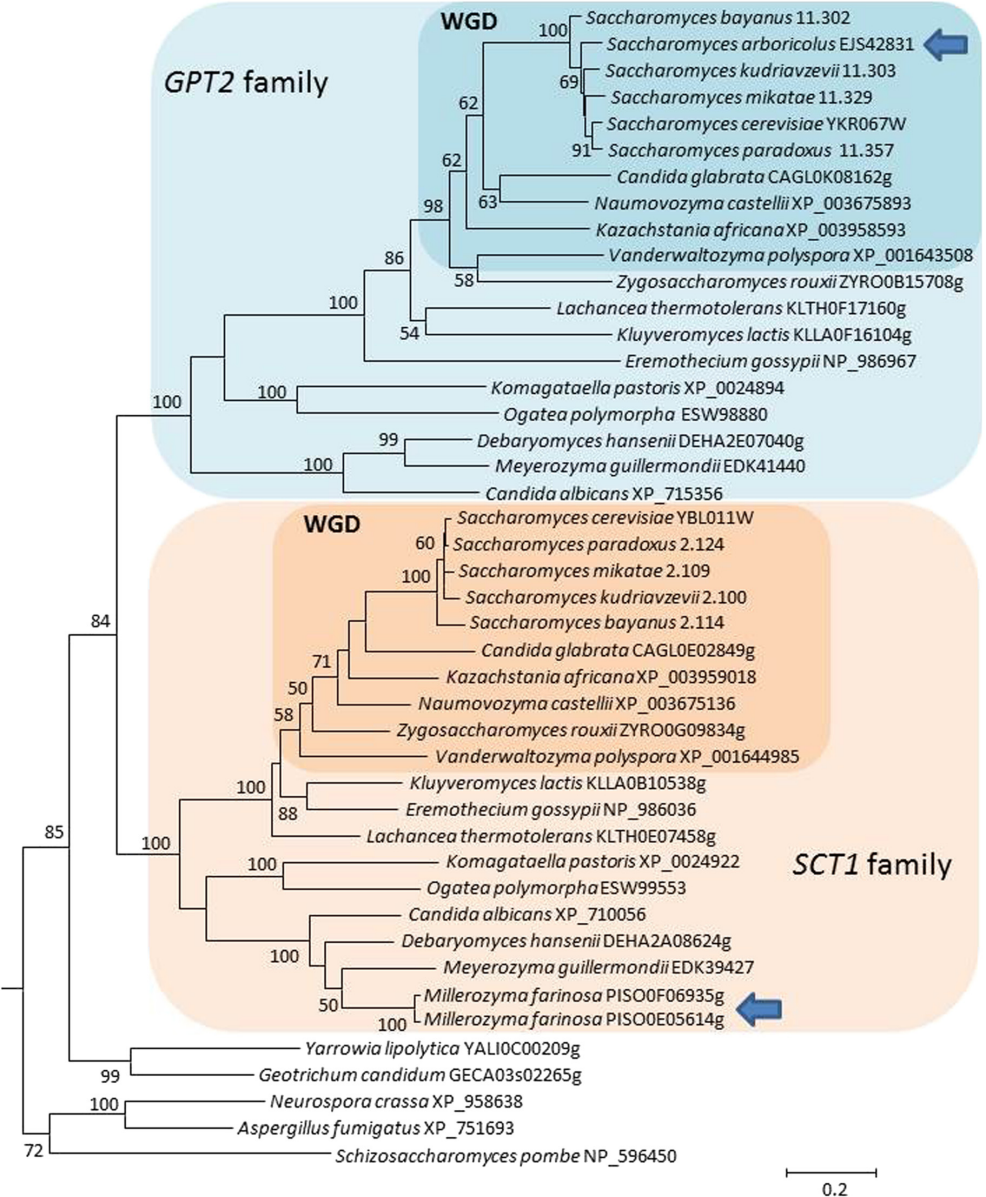
Phylogenetic analysis of the homologs of the *SCT1* and *GPT2* genes in various species. The evolutionary history was inferred by using the maximum-likelihood method based on the Whelan and Goldman model. The tree with the highest log likelihood (−25184.6904) is shown. The percentage of trees in which the same taxa shared a given node is indicated; only values over 50 % are shown. A discrete Gamma distribution was used to model evolutionary rate differences among sites (5 categories (+G, parameter = 1.6929)). The analysis involved 45 amino acid sequences. All positions containing gaps and missing data were eliminated. There were a total of 516 positions in the final dataset. Evolutionary analyses were conducted in MEGA6 (Additional file 2). The basidiomycete *Cryptococccus neoformans* sequence XP_569487 was used as an outgroup. Scale bar, 20 per 100 substitutions per site. The *SCT1* and *GPT2* gene families are boxed. WGD species are indicated. The two species with only one gene copy are indicated by an arrow. Note that *Zygosaccharomyces rouxii* is a non-WGD species, but phylogenetic position derived from single gene analysis may be inaccurate for some species

Our phylogenetic analysis uncovered an ancestral duplication that gave rise to the *SCT1* and *GPT2* gene families in the Saccharomycotina subphylum (Fig. 8). This duplication did not include the species of the family Dipodascaceae (*Yarrowia lipolytica* and *Geotrichum candidum*), nor any of the basal fungal genomes tested such as *Schizosaccharomyces pombe*, *Neurospora crassa*, *Aspergillus fumigatus* and *Cryptococcus neoformans* (Table 4, Fig. 8 and Additional file 6: Figure S2). The tree in Fig. 8 also indicates accelerated evolution of the *GPT2* gene family compared with the *SCT1* gene family. For example, the average branch length of the *GPT2* genes was 49.8 % longer than that of the *SCT1* genes with values of 0.99 and 0.66 substitutions per site, respectively. Original behavior was observed for *MUM3* because the gene was absent in the Dipodascaceae family and other analyzed Ascomycota, suggesting that it may have been acquired recently, after the divergence of the Dipodascaceae from the rest of the Saccharomycotina phylum. Despite the variable distribution of the genes analyzed here, the observed differences in FA production in all the species examined, especially MCFA, was not correlated with the presence/absence of any of the examined genes. Although there is a clear difference between pre- and post-WGD species in terms of FA production, gene content did not reflect this difference. Among the *Saccharomyces* species analyzed, only the genome of *S. arboricolus* has lost genes of interest. These include *SCT1*, *CST26*, *YDR018C* and *MUM3*. However, there was no obvious difference in FA production when *S. arboricolus* was compared with the other species of the genus *Saccharomyces* or the other post-WGD species. In particular, the lack of *SCT1* did not result in a differential production of any FAs. This suggests that there is gene compensation in the species that lack these genes such as *S. arboricolus*, or that have only two copies of the same gene, such as *M. farinosa* for *SCT1.*

## Discussion

In this study, we investigated the MCFA content in various yeast species. To date, this class of lipids has been little studied because these FAs are minor in *S. cerevisiae* and absent from oleaginous yeasts commonly used for tailored lipid production. Furthermore, MCFAs have probably been disregarded because they can be lost during extraction due to their high volatility. For these reasons, it is important to design procedures that avoid evaporation during extraction and that use splitless injection during GC analysis. However, due to their value as substitutes for petroleum derivatives, MCFAs and their production in yeasts is an emerging research topic [4]. Efficient lipid extraction procedures have therefore been developed and it is now possible to explore the MCFA content in *S. cerevisiae* and other Saccharomycotina species.

Testing various culture conditions on FA profiles, we demonstrated that MCFAs preferentially accumulate in WT and mutant *S. cerevisiae* when cells are grown on high-N synthetic media. In most cases, low temperature (23 °C) had a positive effect on MCFA content (Additional file 4: Table S1) due to higher total FA content (Table 2), as confirmed for *S. uvarum*. Furthermore, MCFAs preferentially accumulated during the early exponential growth phase for two post-WGD species. Until recently, applied biotechnology and metabolic engineering has focused on improving lipid accumulation in cells. However, new lipidome approaches show that culture conditions greatly influence lipid production and FA composition [14], although FA chain length has not been carefully investigated. Our results confirmed these observations and showed that culture conditions influence MCFA content; investigating culture conditions is thus an important factor for the targeted production of desired fatty acids.

We also screened *S. cerevisiae* mutant cells with defects in proteins associated with LDs. Two of the analyzed mutants, *loa1*Δ and *tgl3*Δ, showed a modified FA profile and an increase in MCFA content. The absence of these lipid metabolism enzymes in LDs affects the FA profiles in lipids, polar (mostly PLs) or nonpolar (mostly TAGs and SEs). Accumulation of MCFAs has been observed in *loa1*Δ cells [16], although they were not explicitly mentioned [47]. In addition, there is a reported increase in saturated C14:0 and C16:0 FAs in TAG fractions from *tgl3*Δ cells, although shorter MCFAs were not analyzed [47]. Here, for the first time, we explored MCFA content in mutants with defective lipid droplets and altered lipid metabolism. Our observations indicate that genetic engineering on endogenous genes can be performed in *S. cerevisiae* to increase MCFA production. Recent studies have employed heterologous expression of mammal, plant, oleaginous yeast or bacteria enzymes in *S. cerevisiae* [17, 54]. The combination of two approaches, endogenous gene modifications and heterologous expression of pertinent enzymes, thus appears to be a promising approach for developing MCFA production in *S. cerevisiae*.

Another way to improve the production of MCFA in yeast is to screen other Saccharomycotina species, some of which may be naturally rich in MCFAs. Our FA profiling on Saccharomycotina yeast revealed that (i) MCFAs only accumulate in post-WGD species and (ii) MCFA content and profile vary greatly among the species analyzed. In addition, PUFA content varied greatly among the Saccharomycotina species. The presence of PUFAs is negatively correlated with the presence of MCFAs. Thus, as observed for MCFAs, research on PUFAs in Saccharomycotina species holds great promise for lipid biotechnology. This is the main focus of another study in progress.

The mechanisms behind this high FA diversity remain to be elucidated, because we could not observe in our gene gain/loss analysis any correlation between gene distribution and FA production. Nevertheless, most of our observations appeared to be associated with the WGD event. WGD is known to have affected many aspects of yeast cell life, including neo-functionalization of duplicated genes and major changes in overall transcription [55–60]. We therefore suggest two potential sources of this variability. First, given the high gene copy conservation in lipid metabolism enzymes among Saccharomycotina yeasts, as revealed in our genomic study, phenotypic variability may be due to the differential regulation of FA metabolism at the transcriptional and post-transcriptional level, due to WGD. For example, *S. arboricolus* showed similar FA production compared with the other Saccharomycotina species despite the fact that its genome lacks four genes, including *SCT1.* Second, MCFAs are specific to post-WGD species, indicating that the WGD may have affected lipid metabolism genes and their functions. The function of some homologs may have evolved within some Saccharomycotina species. For example, Are1p and Are2p acyltransferases [61] and TAG lipases play different roles in *Y. lipolytica* compared to their homologs in other yeast species. In *Y. lipolytica*, the main TAG lipase activity is carried out by YlTgl4p and not byYlTgl3p, the homolog of the main *S. cerevisiae* TAG lipase [62]. In addition, *YDR018c*, encoding a LPLAT family enzyme, is only present in *Saccharomyces* species. Interestingly, all lipid enzyme classes contain genes that have been duplicated during the WGD and some may potentially have some lipid metabolism functions specific to post-WGD species. It is possible to extend screening to find new strains with relevant MCFA profiles. For example, *S. uvarum* and *S. bayanus* appear to have high MFCA content. Our FA analysis revealed that *S. uvarum* contains 9.2 % MCFAs when grown at 28 °C. Furthermore, one study shows that *S. bayanus* produces up to 36 % MCFAs when grown on white must at 13 °C [63]. Thus, there is a relationship between phenotype and the ecological and geographical origin of strains, in particular for lipid and FA composition. Numerous strains and natural interspecific *Saccharomyces* hybrids isolated from low temperature fermentation or from cold regions are available in yeast collections. FA profiling of these strains after cultivation at favorable temperatures (10–15 °C) can potentially lead to the discovery of specific high-performance cells or FA enzymes useful for MCFA production for biotechnology purposes.

## Conclusions

In this study, we explored the MCFA content and production in Saccharomycotina yeasts with regard to the few reports in the literature on MCFA production by microorganisms despite their biotechnological interest. First, MCFA content in *S. cerevisiae* varies according to culture conditions and to lipid class. MCFAs are more abundant in neutral lipids than in phospholipids. Using genetic screening, we identified two genes, *LOA1* and *TGL3*, involved in MCFA homeostasis. These results demonstrate that yeast engineering holds promise as an approach for developing MCFA production in *S. cerevisiae*. Second, FA profiling in 16 Saccharomycotina species showed that they have contrasting phenotypes that follow a phylogenetic pattern. We demonstrated that lipid-containing MCFAs are only present in post-WGD yeasts and revealed that Saccharomycotina yeasts are promising biological resources for biotechnology.

### Availability of supporting data

The data sets supporting the results of this article are included within the article and its Additional files 2 and 3, Additional file 1: Figure S1; Additional file 6: Figure S2; Additional file 7: Figure S3; Additional file 8: Figure S4; Additional file 9: Figure S5; Additional file 10: Figure S6; Additional file 11: Figure S7; Additional file 12: Figure S8 and Additional file 4: Table S1; Additional file 5: Table S2.

## Additional files

Additional file 1: Figure S1. Growth curves of strains used in this study. Growth curves of *Saccharomyces cerevisiae* BY4741 WT, *loa1Δ* and *tgl3Δ* cells grown in YP, high- or low-N YNB at 23 °C (A) and 28 °C (B) and *S. cerevisiae* S288C and *Saccharomyces uvarum* grown in YP at 23 °C (C) and 28 °C (D).

Additional file 2: **MEGA6 multiple sequence alignment of the proteins encoded by the** ***SCT1*** **and** ***GPT2*** **gene family used for Figure** 8 **tree.**

Additional file 3: **MAFFT multiple sequence alignments of all the proteins studied.**

Additional file 4: Table S1. Fatty acid content in the wild-type (WT), *tgl3*Δ and *loa1*Δ strains grown in various conditions. FAME, fatty acid methyl ester.

Additional file 5: Table S2. Accession numbers of LPLAT genes in 26 yeast species. Accession numbers as assigned in the Saccharomyces Genome Database (SGD)^1^, Scannel *et al.* [37]^2^ , the National Center for Biotechnology Information (NCBI)^3^, the Génolevures Database^4^, the Candida GenomeDatabase (CGD)^5^ or the Online Resource for Community Annotation of Eukaryotes (ORCAE)^6^.

Additional file 6: Figure S2. Multiple sequence alignment of the proteins encoded by the *SCT1* and *GPT2* gene family. Multiple sequence alignments of the proteins encoded by the LPLAT genes. Sequence alignment was generated using MAFFT ver. 7 and visualized using Jalview 2 [65]. Each residue in the alignment was assigned a color (ClustalX Color Scheme) if the amino acid profile of the alignment at that position meets some minimum criteria specific for the residue type (for details, see http://www.jalview.org/help/html/colourSchemes/clustal.html).

Additional file 7: Figure S3. Multiple sequence alignments of the proteins encoded by *CST26 and YDR018c* gene family. Multiple sequence alignments of the proteins encoded by the LPLAT genes. Sequence alignment was generated using MAFFT ver. 7 and visualized using Jalview 2 [65]. Each residue in the alignment was assigned a color (ClustalX Color Scheme) if the amino acid profile of the alignment at that position meets some minimum criteria specific for the residue type (for details, see http://www.jalview.org/help/html/colourSchemes/clustal.html).

Additional file 8: Figure S4. Multiple sequence alignments of the proteins encoded by *SLC1* genes. Multiple sequence alignments of the proteins encoded by the LPLAT genes. Sequence alignment was generated using MAFFT ver. 7 and visualized using Jalview 2 [65]. Each residue in the alignment was assigned a color (ClustalX Color Scheme) if the amino acid profile of the alignment at that position meets some minimum criteria specific for the residue type (for details, see http://www.jalview.org/help/html/colourSchemes/clustal.html).

Additional file 9: Figure S5. Multiple sequence alignments of the proteins encoded by *DGA1* genes. Multiple sequence alignments of the proteins encoded by the LPLAT genes. Sequence alignment was generated using MAFFT ver. 7 and visualized using Jalview 2 [65]. Each residue in the alignment was assigned a color (ClustalX Color Scheme) if the amino acid profile of the alignment at that position meets some minimum criteria specific for the residue type (for details, see http://www.jalview.org/help/html/colourSchemes/clustal.html).

Additional file 10: Figure S6. Multiple sequence alignments of the proteins encoded by *TAZ1* genes. Multiple sequence alignments of the proteins encoded by the LPLAT genes. Sequence alignment was generated using MAFFT ver. 7 and visualized using Jalview 2 [65]. Each residue in the alignment was assigned a color (ClustalX Color Scheme) if the amino acid profile of the alignment at that position meets some minimum criteria specific for the residue type (for details, see http://www.jalview.org/help/html/colourSchemes/clustal.html).

Additional file 11: Figure S7. Multiple sequence alignments of the proteins encoded by *LOA1* genes. Multiple sequence alignments of the proteins encoded by the LPLAT genes. Sequence alignment was generated using MAFFT ver. 7 and visualized using Jalview 2 [65]. Each residue in the alignment was assigned a color (ClustalX Color Scheme) if the amino acid profile of the alignment at that position meets some minimum criteria specific for the residue type (for details, see http://www.jalview.org/help/html/colourSchemes/clustal.html).

Additional file 12: Figure S8. Multiple sequence alignments of the proteins encoded by *MUM3* genes. Multiple sequence alignments of the proteins encoded by the LPLAT genes. Sequence alignment was generated using MAFFT ver. 7 and visualized using Jalview 2 [65]. Each residue in the alignment was assigned a color (ClustalX Color Scheme) if the amino acid profile of the alignment at that position meets some minimum criteria specific for the residue type (for details, see http://www.jalview.org/help/html/colourSchemes/clustal.html).

## Abbreviations

DAG: Diacylglycerol
FA: Fatty acid
FAME: Fatty acid methyl ester
FID: Flame ionization detection
GC: Gas chromatography
LD: Lipid droplet
LPLAT: Lysophospholipid acylytransferase
MCFA: Medium-chain fatty acid
PUFA: Polyunsaturated fatty acid
SE: Sterol ester
TLC: Thin layer chromatography
WGD: Whole genome duplication

## References

[1] Dyer JM, Stymne S, Green AG, Carlsson AS (2008). High-value oils from plants. Plant J.

[2] Hill K, Kamm B, Gruber PR, Kamm M (2006). Industrial Development and Application of Biobased Oleochemicals. Biorefineries—Industrial Processes and Products Status Quo and Future Directions, vol. 2.

[3] Gervajio GC, Shahidi F (2005). Fatty Acids and Derivatives from Coconut Oil. Bailey’s Industrial Oil and Fat Products, Sixth Edition, vol. 6.

[4] Lee SK, Chou H, Ham TS, Lee TS, Keasling JD (2008). Metabolic engineering of microorganisms for biofuels production: from bugs to synthetic biology to fuels. Curr Opin Biotechnol.

[5] Pinzi S, Garcia IL, Lopez Gimenez FJ, Luque de Castro MD, Dorado G, Dorado MP (2009). The ideal vegetable oil–based biodiesel composition: a review of social, economical and technical implications. Energy Fuels.

[6] Tjellstrom H, Strawsine M, Silva J, Cahoon EB, Ohlrogge JB (2013). Disruption of plastid acyl:acyl carrier protein synthetases increases medium chain fatty acid accumulation in seeds of transgenic Arabidopsis. FEBS Lett.

[7] Hu Q, Sommerfeld M, Qin S (2012). Algal Medium-Chain Lenght Fatty Acids and Hydrocarbons. vol. US 20120135478 A1: Arizone Board of Regents for and on Behalf of Arizona State university.

[8] Grootscholten TI, Steinbusch KJ, Hamelers HV, Buisman CJ (2012). Chain elongation of acetate and ethanol in an upflow anaerobic filter for high rate MCFA production. Bioresour Technol.

[9] Torella JP, Ford TJ, Kim SN, Chen AM, Way JC, Silver PA (2013). Tailored fatty acid synthesis via dynamic control of fatty acid elongation. Proc Natl Acad Sci U S A.

[10] Parfene G, Horincar V, Tyagi AK, Malik A, Bahrim G (2013). Production of medium chain saturated fatty acids with enhanced antimicrobial activity from crude coconut fat by solid state cultivation of Yarrowia lipolytica. Food Chem.

[11] Redon M, Guillamon JM, Mas A, Rozes N (2011). Effect of growth temperature on yeast lipid composition and alcoholic fermentation at low temperature. Eur Food Res Technol.

[12] Tronchoni J, Rozes N, Querol A, Guillamon JM (2012). Lipid composition of wine strains of Saccharomyces kudriavzevii and Saccharomyces cerevisiae grown at low temperature. Int J Food Microbiol.

[13] Ejsing CS, Sampaio JL, Surendranath V, Duchoslav E, Ekroos K, Klemm RW (2009). Global analysis of the yeast lipidome by quantitative shotgun mass spectrometry. Proc Natl Acad Sci U S A.

[14] Klose C, Surma MA, Gerl MJ, Meyenhofer F, Shevchenko A, Simons K (2012). Flexibility of a eukaryotic lipidome–insights from yeast lipidomics. PLoS One.

[15] Grillitsch K, Connerth M, Kofeler H, Arrey TN, Rietschel B, Wagner B (2011). Lipid particles/droplets of the yeast Saccharomyces cerevisiae revisited: lipidome meets proteome. Biochim Biophys Acta.

[16] Ayciriex S, Le Guedard M, Camougrand N, Velours G, Schoene M, Leone S (2012). YPR139c/LOA1 encodes a novel lysophosphatidic acid acyltransferase associated with lipid droplets and involved in TAG homeostasis. Mol Biol Cell.

[17] Chen L, Zhang J, Chen WN (2014). Engineering the Saccharomyces cerevisiae beta-oxidation pathway to increase medium chain fatty acid production as potential biofuel. PLoS One.

[18] Johnson EA (2013). Biotechnology of non-Saccharomyces yeasts-the ascomycetes. Appl Microbiol Biotechnol.

[19] Kurtzman CP, Fell JW, Boekhout T (2011). The Yeasts, A Taxonomic Study.

[20] Weiss S, Samson F, Navarro D, Casaregola S (2013). YeastIP: a database for identification and phylogeny of Saccharomycotina yeasts. FEMS Yeast Res.

[21] James TY, Kauff F, Schoch CL, Matheny PB, Hofstetter V, Cox CJ (2006). Reconstructing the early evolution of Fungi using a six-gene phylogeny. Nature.

[22] Rolland T, Dujon B (2011). Yeasty clocks: dating genomic changes in yeasts. C R Biol.

[23] Wolfe KH, Shields DC (1997). Molecular evidence for an ancient duplication of the entire yeast genome. Nature.

[24] Ratledge C (2004). Fatty acid biosynthesis in microorganisms being used for Single Cell Oil production. Biochimie.

[25] Wynn JP, Ratledge C, Shahidi F (2005). Oils from Microorganisms. Bailey’s Industrial Oil and Fat Products, Sixth Edition, vol. 3.

[26] Beopoulos A, Cescut J, Haddouche R, Uribelarrea JL, Molina-Jouve C, Nicaud JM (2009). Yarrowia lipolytica as a model for bio-oil production. Prog Lipid Res.

[27] Azocar L, Ciudad G, Heipieper HJ, Navia R (2010). Biotechnological processes for biodiesel production using alternative oils. Appl Microbiol Biotechnol.

[28] Froissard M, D’Andrea S, Boulard C, Chardot T (2009). Heterologous expression of AtClo1, a plant oil body protein, induces lipid accumulation in yeast. FEMS Yeast Res.

[29] Debelyy MO, Thoms S, Connerth M, Daum G, Erdmann R (2011). Involvement of the Saccharomyces cerevisiae hydrolase Ldh1p in lipid homeostasis. Eukaryot Cell.

[30] Fei W, Shui G, Zhang Y, Krahmer N, Ferguson C, Kapterian TS (2011). A role for phosphatidic acid in the formation of “supersized” lipid droplets. PLoS Genet.

[31] Yu KO, Jung J, Ramzi AB, Choe SH, Kim SW, Park C (2013). Development of a Saccharomyces cerevisiae strain for increasing the accumulation of triacylglycerol as a microbial oil feedstock for biodiesel production using glycerol as a substrate. Biotechnol Bioeng.

[32] Janke C, Magiera MM, Rathfelder N, Taxis C, Reber S, Maekawa H (2004). A versatile toolbox for PCR-based tagging of yeast genes: new fluorescent proteins, more markers and promoter substitution cassettes. Yeast.

[33] Folch J, Lees M, Sloane Stanley GH (1957). A simple method for the isolation and purification of total lipides from animal tissues. J Biol Chem.

[34] Schneiter R, Daum G (2006). Extraction of yeast lipids. Methods Mol Biol.

[35] Athenstaedt K, Zweytick D, Jandrositz A, Kohlwein SD, Daum G (1999). Identification and characterization of major lipid particle proteins of the yeast Saccharomyces cerevisiae. J Bacteriol.

[36] Schneiter R, Daum G (2006). Analysis of yeast lipids. Methods Mol Biol.

[37] Scannell DR, Zill OA, Rokas A, Payen C, Dunham MJ, Eisen MB (2011). The awesome power of yeast evolutionary genetics: new genome sequences and strain resources for the Saccharomyces sensu stricto genus. G3 (Bethesda).

[38] Dereeper A, Guignon V, Blanc G, Audic S, Buffet S, Chevenet F (2008). Phylogeny.fr: robust phylogenetic analysis for the non-specialist. Nucleic Acids Res.

[39] Whelan S, Goldman N (2001). A general empirical model of protein evolution derived from multiple protein families using a maximum-likelihood approach. Mol Biol Evol.

[40] Tamura K, Stecher G, Peterson D, Filipski A, Kumar S (2013). MEGA6: molecular evolutionary genetics analysis version 6.0. Mol Biol Evol.

[41] Perriere G, Gouy M (1996). WWW-query: an on-line retrieval system for biological sequence banks. Biochimie.

[42] Brasaemle DL, Wolins NE (2012). Packaging of fat: an evolving model of lipid droplet assembly and expansion. J Biol Chem.

[43] Chapman KD, Dyer JM, Mullen RT (2012). Biogenesis and functions of lipid droplets in plants: thematic review series: lipid droplet synthesis and metabolism: from yeast to man. J Lipid Res.

[44] Murphy DJ (2012). The dynamic roles of intracellular lipid droplets: from archaea to mammals. Protoplasma.

[45] Walther TC, Farese RV (2012). Lipid droplets and cellular lipid metabolism. Annu Rev Biochem.

[46] Athenstaedt K, Jolivet P, Boulard C, Zivy M, Negroni L, Nicaud JM (2006). Lipid particle composition of the yeast Yarrowia lipolytica depends on the carbon source. Proteomics.

[47] Athenstaedt K, Daum G (2003). YMR313c/TGL3 encodes a novel triacylglycerol lipase located in lipid particles of Saccharomyces cerevisiae. J Biol Chem.

[48] Rajakumari S, Daum G (2010). Janus-faced enzymes yeast Tgl3p and Tgl5p catalyze lipase and acyltransferase reactions. Mol Biol Cell.

[49] Beopoulos A, Nicaud JM, Gaillardin C (2011). An overview of lipid metabolism in yeasts and its impact on biotechnological processes. Appl Microbiol Biotechnol.

[50] Ivashov VA, Grillitsch K, Koefeler H, Leitner E, Baeumlisberger D, Karas M (2013). Lipidome and proteome of lipid droplets from the methylotrophic yeast Pichia pastoris. Biochim Biophys Acta.

[51] Kainou K, Kamisaka Y, Kimura K, Uemura H (2006). Isolation of Delta12 and omega3-fatty acid desaturase genes from the yeast Kluyveromyces lactis and their heterologous expression to produce linoleic and alpha-linolenic acids in Saccharomyces cerevisiae. Yeast.

[52] Louis VL, Despons L, Friedrich A, Martin T, Durrens P, Casaregola S (2012). Pichia sorbitophila, an interspecies yeast hybrid, reveals early steps of genome resolution after polyploidization. G3 (Bethesda).

[53] Langkjaer RB, Cliften PF, Johnston M, Piskur J (2003). Yeast genome duplication was followed by asynchronous differentiation of duplicated genes. Nature.

[54] Leber C, Da Silva NA (2014). Engineering of Saccharomyces cerevisiae for the synthesis of short chain fatty acids. Biotechnol Bioeng.

[55] Conant GC, Wolfe KH (2008). Turning a hobby into a job: how duplicated genes find new functions. Nat Rev Genet.

[56] Presser A, Elowitz MB, Kellis M, Kishony R (2008). The evolutionary dynamics of the Saccharomyces cerevisiae protein interaction network after duplication. Proc Natl Acad Sci U S A.

[57] Scannell DR, Wolfe KH (2008). A burst of protein sequence evolution and a prolonged period of asymmetric evolution follow gene duplication in yeast. Genome Res.

[58] van Hoek MJ, Hogeweg P (2009). Metabolic adaptation after whole genome duplication. Mol Biol Evol.

[59] Conant GC (2010). Rapid reorganization of the transcriptional regulatory network after genome duplication in yeast. Proc Biol Sci.

[60] Fares MA, Keane OM, Toft C, Carretero-Paulet L, Jones GW (2013). The roles of whole-genome and small-scale duplications in the functional specialization of Saccharomyces cerevisiae genes. PLoS Genet.

[61] Beopoulos A, Haddouche R, Kabran P, Dulermo T, Chardot T, Nicaud JM (2012). Identification and characterization of DGA2, an acyltransferase of the DGAT1 acyl-CoA:diacylglycerol acyltransferase family in the oleaginous yeast Yarrowia lipolytica. New insights into the storage lipid metabolism of oleaginous yeasts. Appl Microbiol Biotechnol.

[62] Dulermo T, Treton B, Beopoulos A, Kabran Gnankon AP, Haddouche R, Nicaud JM (2013). Characterization of the two intracellular lipases of Y. lipolytica encoded by TGL3 and TGL4 genes: new insights into the role of intracellular lipases and lipid body organisation. Biochim Biophys Acta.

[63] Torija MJ, Beltran G, Novo M, Poblet M, Guillamon JM, Mas A (2003). Effects of fermentation temperature and Saccharomyces species on the cell fatty acid composition and presence of volatile compounds in wine. Int J Food Microbiol.

[64] Dujon B (2010). Yeast evolutionary genomics. Nat Rev Genet.

[65] Waterhouse AM, Procter JB, Martin DM, Clamp M, Barton GJ (2009). Jalview Version 2—a multiple sequence alignment editor and analysis workbench. Bioinformatics.

