# Supplementary figures and images for: Lipids containing medium-chain fatty acids are specific to post-whole genome duplication Saccharomycotina yeasts
Source: BMC Evol Biol. 2015 May 28;15:97. doi: 10.1186/s12862-015-0369-2 (PMC4446107; doi:10.1186/s12862-015-0369-2)

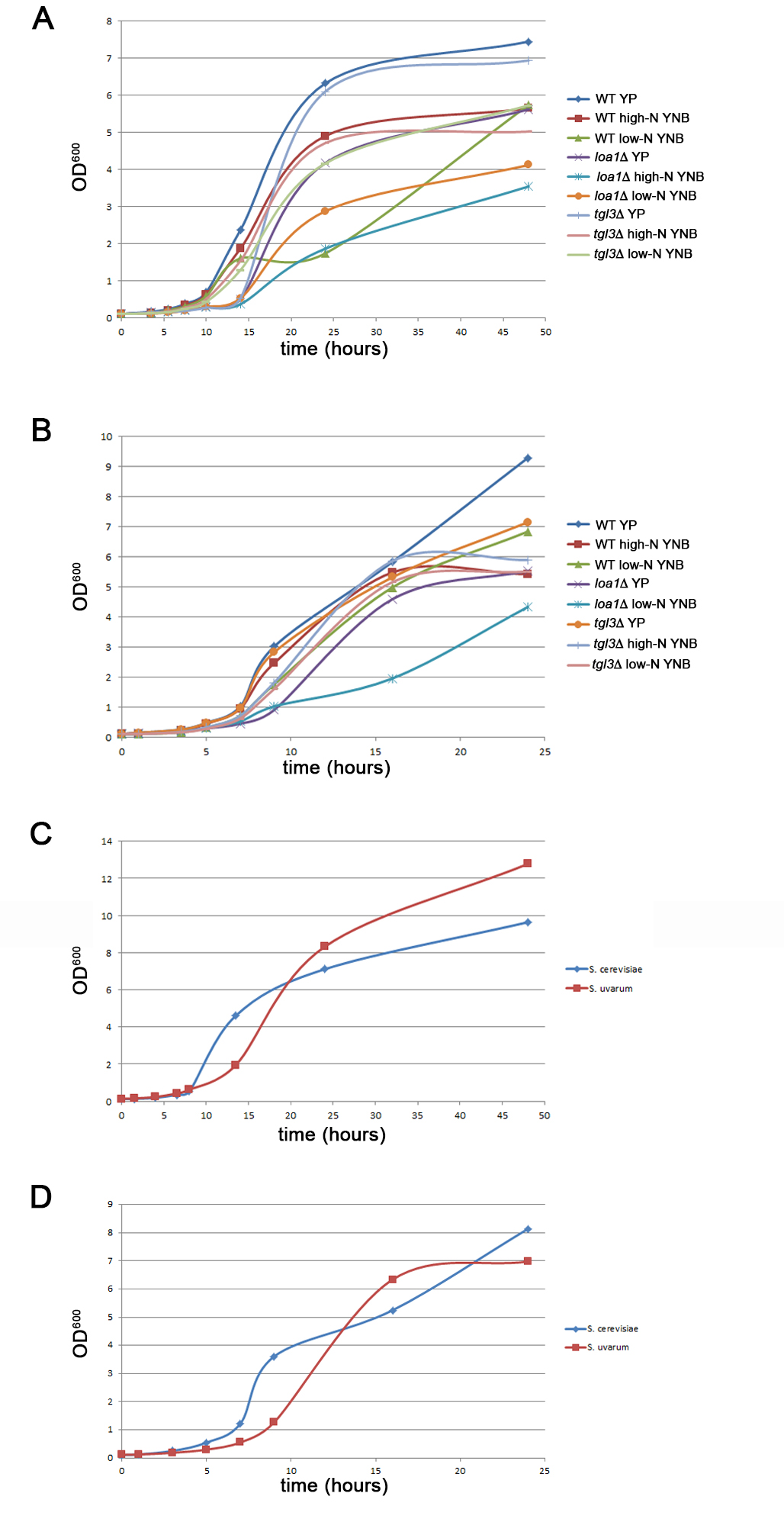

Supplement: Supplementary file 1 — Growth curves of strains used in this study. Growth curves of Saccharomyces cerevisiae BY4741 WT, loa1Δ and tgl3Δ cells grown in YP, high- or low-N YNB at 23 °C (A) and 28 °C (B) and S. cerevisiae S288C and Saccharomyces uvarum grown in YP at 23 °C (C) and 28 °C (D). [file 12862_2015_369_MOESM1_ESM.jpeg]

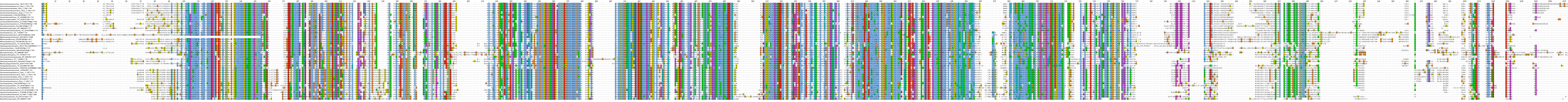

Supplement: Supplementary file 6 — Multiple sequence alignment of the proteins encoded by the SCT1 and GPT2 gene family. Multiple sequence alignments of the proteins encoded by the LPLAT genes. Sequence alignment was generated using MAFFT ver. 7 and visualized using Jalview 2 [65]. Each residue in the alignment was assigned a color (ClustalX Color Scheme) if the amino acid profile of the alignment at that position meets some minimum criteria specific for the residue type (for details, see http://www.jalview.org/help/html/colourSchemes/clustal.html). [file 12862_2015_369_MOESM6_ESM.png]

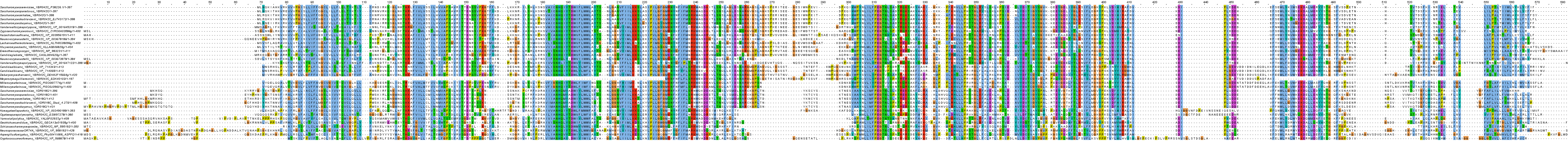

Supplement: Supplementary file 7 — Multiple sequence alignments of the proteins encoded by CST26 and YDR018c gene family. Multiple sequence alignments of the proteins encoded by the LPLAT genes. Sequence alignment was generated using MAFFT ver. 7 and visualized using Jalview 2 [65]. Each residue in the alignment was assigned a color (ClustalX Color Scheme) if the amino acid profile of the alignment at that position meets some minimum criteria specific for the residue type (for details, see http://www.jalview.org/help/html/colourSchemes/clustal.html). [file 12862_2015_369_MOESM7_ESM.png]

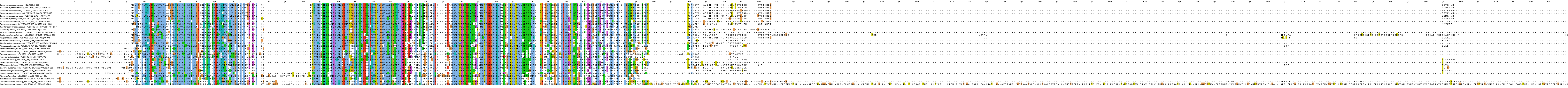

Supplement: Supplementary file 8 — Multiple sequence alignments of the proteins encoded by SLC1 genes. Multiple sequence alignments of the proteins encoded by the LPLAT genes. Sequence alignment was generated using MAFFT ver. 7 and visualized using Jalview 2 [65]. Each residue in the alignment was assigned a color (ClustalX Color Scheme) if the amino acid profile of the alignment at that position meets some minimum criteria specific for the residue type (for details, see http://www.jalview.org/help/html/colourSchemes/clustal.html). [file 12862_2015_369_MOESM8_ESM.png]

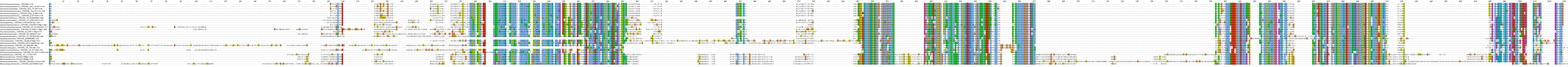

Supplement: Supplementary file 9 — Multiple sequence alignments of the proteins encoded by DGA1 genes. Multiple sequence alignments of the proteins encoded by the LPLAT genes. Sequence alignment was generated using MAFFT ver. 7 and visualized using Jalview 2 [65]. Each residue in the alignment was assigned a color (ClustalX Color Scheme) if the amino acid profile of the alignment at that position meets some minimum criteria specific for the residue type (for details, see http://www.jalview.org/help/html/colourSchemes/clustal.html). [file 12862_2015_369_MOESM9_ESM.png]

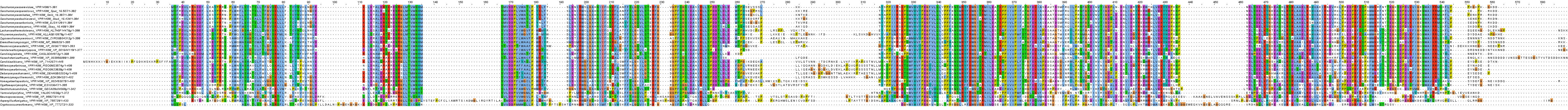

Supplement: Supplementary file 10 — Multiple sequence alignments of the proteins encoded by TAZ1 genes. Multiple sequence alignments of the proteins encoded by the LPLAT genes. Sequence alignment was generated using MAFFT ver. 7 and visualized using Jalview 2 [65]. Each residue in the alignment was assigned a color (ClustalX Color Scheme) if the amino acid profile of the alignment at that position meets some minimum criteria specific for the residue type (for details, see http://www.jalview.org/help/html/colourSchemes/clustal.html). [file 12862_2015_369_MOESM10_ESM.png]

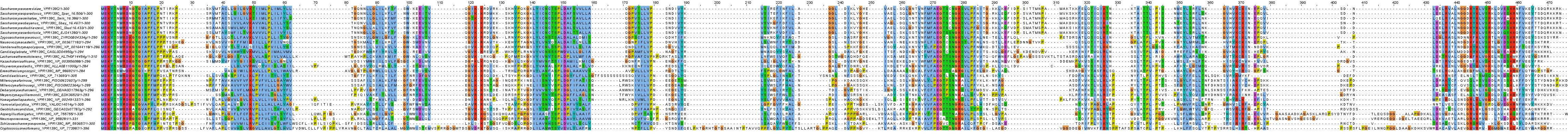

Supplement: Supplementary file 11 — Multiple sequence alignments of the proteins encoded by LOA1 genes. Multiple sequence alignments of the proteins encoded by the LPLAT genes. Sequence alignment was generated using MAFFT ver. 7 and visualized using Jalview 2 [65]. Each residue in the alignment was assigned a color (ClustalX Color Scheme) if the amino acid profile of the alignment at that position meets some minimum criteria specific for the residue type (for details, see http://www.jalview.org/help/html/colourSchemes/clustal.html). [file 12862_2015_369_MOESM11_ESM.png]

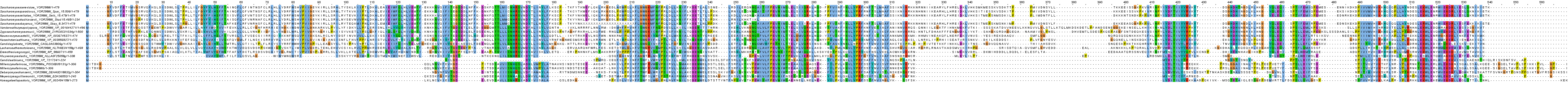

Supplement: Supplementary file 12 — Multiple sequence alignments of the proteins encoded by MUM3 genes. Multiple sequence alignments of the proteins encoded by the LPLAT genes. Sequence alignment was generated using MAFFT ver. 7 and visualized using Jalview 2 [65]. Each residue in the alignment was assigned a color (ClustalX Color Scheme) if the amino acid profile of the alignment at that position meets some minimum criteria specific for the residue type (for details, see http://www.jalview.org/help/html/colourSchemes/clustal.html). [file 12862_2015_369_MOESM12_ESM.png]
