# Supplementary material for: Lipids containing medium-chain fatty acids are specific to post-whole genome duplication Saccharomycotina yeasts
Source: BMC Evol Biol. 2015 May 28;15:97. doi: 10.1186/s12862-015-0369-2 (PMC4446107; doi:10.1186/s12862-015-0369-2)
Supplement: Supplementary file 4 — Fatty acid content in the wild-type (WT), tgl3Δ and loa1Δ strains grown in various conditions. FAME, fatty acid methyl ester. [file 12862_2015_369_MOESM4_ESM.doc]

Supplementary Table S1: Fatty acid content in the wild-type (WT), *tgl3*Δ and *loa1*Δ strains grown in various conditions. FAME, fatty acid methyl ester

| Strain | Growth medium | Temperature | Fatty acids (µg FAME.mg^-1^ dry weight) | | | | | | | | |  |
| --- | --- | --- | --- | --- | --- | --- | --- | --- | --- | --- | --- | --- |
|  |  | (°C) | C8:0 | C10:0 | C12:0 | C14:0 | C14:1 | C16:0 | C16:1 | C18:0 | C18:1 | Total MCFA  (C8:0 –C14:0) |
| WT | YP | 23 | 0.00 | 1.59 ± 0.48 | 1.21 ± 0.27 | 0.90 ± 0.11 | 0.47 ± 0.09 | 8.01 ± 0.37 | 28.81 ± 2.80 | 1.87 ± 0.02 | 12.08 ± 0.48 | 3.69 ± 0.87 |
| WT | YP | 28 | 0.00 | 0.90 ± 0.07 | 0.82 ± 0.06 | 0.57 ± 0.03 | 0.21 ± 0.01 | 7.37 ± 0.27 | 21.98 ± 0.98 | 1.75 ± 0.06 | 11.45 ± 0.40 | 2.29 ± 0.15 |
| WT | High-N YNB | 23 | 0.27 ± 0.13 | 2.63 ± 1.22 | 1.60 ± 0.56 | 1.30 ± 0.22 | 0.50 ± 0.14 | 9.43 ± 0.52 | 26.16 ± 3.12 | 2.35 ± 0.03 | 11.90 ± 0.09 | 5.79 ± 2.13 |
| WT | High-N YNB | 28 | 0.29 0.± 0.08 | 3.04 ± 0.78 | 1.85 ± 0.23 | 1.06 ± 0.04 | 0.35 ± 0.03 | 9.33 ± 1.01 | 22.50 ± 1.44 | 2.43 ± 0.31 | 11.52 ± 1.38 | 6.24 ± 1.13 |
| WT | Low-N YNB | 23 | 0.18 ± 0.01 | 1.65 ± 0.26 | 1.40 ± 0.21 | 1.19 ± 0.05 | 0.36 ± 0.04 | 15.25 ± 0.11 | 25.37 ± 0.55 | 3.71 ± 0.27 | 13.08 ± 0.67 | 4.42 ± 0.53 |
| WT | Low-N YNB | 28 | 0.35 ± 0.08 | 1.87 ± 0.43 | 1.77 ± 0.30 | 1.39 ± 0.09 | 0.35 ± 0.05 | 14.17 ± 0.48 | 21.98 ± 1.09 | 2.39 ± 0.16 | 12.14 ± 0.04 | 5.37 ± 0.90 |
| *tgl3*Δ | YP | 23 | 0.09 ± 0.02 | 3.62 ± 0.87 | 2.40 ± 0.37 | 2.03 ± 0.15 | 0.92 ± 0.11 | 11.46 ± 0.33 | 33.98 ± 1.92 | 2.37 ± 0.03 | 12.79 ± 0.39 | 8.13 ± 1.41 |
| *tgl3*Δ | YP | 28 | 0.04 ± 0.00 | 1.64 ± 0.12 | 1.29 ± 0.05 | 1.26 ± 0.03 | 0.37 ± 0.01 | 10.26 ± 0.21 | 25.32 ± 0.50 | 2.07 ± 0.05 | 12.44 ± 0.26 | 4.23 ± 0.19 |
| *tgl3*Δ | High-N YNB | 23 | 0.27 ± 0.12 | 5.59 ± 2.45 | 3.00 ± 1.14 | 2.90 ± 0.56 | 0.90 ± 0.29 | 13.32 ± 0.74 | 30.52 ± 4.28 | 2.83 ± 0.02 | 12.26 ± 0.41 | 11.76 ± 4.28 |
| *tgl3*Δ | High-N YNB | 28 | 0.68 ± 0.21 | 5.43 ± 1.65 | 2.83 ± 0.69 | 2.14 ± 0.26 | 0.49 ± 0.10 | 12.20 ± 0.73 | 21.60 ± 2.01 | 2.55 ± 0.23 | 9.80 ± 0.51 | 11.07 ± 2.81 |
| *tgl3*Δ | Low-N YNB | 23 | 0.27 ± 0.04 | 2.71 ± 0.48 | 1.89 ± 0.31 | 2.06 ± 0.10 | 0.54 ± 0.07 | 18.33 ± 0.10 | 30.26 ± 0.90 | 4.47 ± 0.08 | 17.02 ± 0.09 | 6.93 ± 0.94 |
| *tgl3*Δ | Low-N YNB | 28 | 0.28 ± 0.04 | 2.09 ± 0.23 | 1.72 ± 0.15 | 2.13 ± 0.03 | 0.40 ± 0.02 | 18.22 ± 0.31 | 27.00 ± 0.29 | 3.54 ± 0.21 | 14.74 ± 0.65 | 6.22 ± 0.45 |
| *loa1*Δ | YP | 23 | 0.09 ± 0.01 | 2.98 ± 0.43 | 2.58 ± 0.32 | 2.10 ± 0.09 | 0.93 ± 0.09 | 9.71 ± 0.05 | 30.08 ± 0.62 | 1.66 ± 0.02 | 10.61 ± 0.10 | 7.74 ± 0.85 |
| *loa1*Δ | YP | 28 | 0.05 ± 0.00 | 2.10 ± 0.08 | 1.86 ± 0.04 | 1.49 ± 0.02 | 0.49 ± 0.01 | 8.64 ± 0.06 | 23.15 ± 0.31 | 1.38 ± 0.01 | 10.04 ± 0.10 | 5.50 ± 0.15 |
| *loa1*Δ | High-N YNB | 23 | 0.30 ± 0.16 | 4.29 ± 1.67 | 2.51 ± 0.74 | 2.10 ± 0.54 | 0.57 ± 0.11 | 10.21 ± 0.75 | 22.87 ± 2.03 | 1.94 ± 0.36 | 8.02 ± 1.51 | 9.20 ± 3.11 |
| *loa1*Δ | High-N YNB | 28 | nd | nd | nd | nd | nd | nd | nd | nd | nd | nd |
| *loa1*Δ | Low-N YNB | 23 | 0.23 ± 0.04 | 2.46 ± 0.39 | 1.93 ± 0.16 | 2.01 ± 0.08 | 0.53 ± 0.03 | 14.92 ± 1.18 | 27.99 ± 1.11 | 4.31 ± 0.04 | 16.18 ± 1.03 | 6.63 ± 0.67 |
| *loa1*Δ | Low-N YNB | 28 | 0.40 ± 0.11 | 2.68 ± 0.75 | 1.98 ± 0.55 | 2.06 ± 0.27 | 0.41 ± 0.10 | 12.34 ± 0.23 | 21.85 ± 2.21 | 2.55 ± 0.18 | 11.16 ± 0.34 | 7.13 ± 1.67 |
