# Supplementary material for: Lipids containing medium-chain fatty acids are specific to post-whole genome duplication Saccharomycotina yeasts
Source: BMC Evol Biol. 2015 May 28;15:97. doi: 10.1186/s12862-015-0369-2 (PMC4446107; doi:10.1186/s12862-015-0369-2)
Supplement: Supplementary file 5 — Accession numbers of LPLAT genes in 26 yeast species. Accession numbers as assigned in the Saccharomyces Genome Database (SGD)1, Scannel et al. [37]2 , the National Center for Biotechnology Information (NCBI)3, the Génolevures Database4, the Candida GenomeDatabase (CGD)5 or the Online Resource for Community Annotation of Eukaryotes (ORCAE)6. [file 12862_2015_369_MOESM5_ESM.doc]

Supplementary Table S2: Accession numbers of LPLAT genes in 26 yeast species.

Accession numbers as assigned in the Saccharomyces Genome Database (SGD)^1^, Scannel *et al.* (2011) ^2^ , the National Center for Biotechnology Information (NCBI)^3^, the Génolevures Database^4^, the Candida GenomeDatabase (CGD)^5^ or the Online Resource for Community Annotation of Eukaryotes (ORCAE)^6^.

| *Species* | *SCT1* | *GPT2* | *CST26* | *YDR018C* | *SLC1* | *DGA1* | *TAZ1* | *LOA1* | *MUM3* |
| --- | --- | --- | --- | --- | --- | --- | --- | --- | --- |
| *Saccharomyces cerevisiae^1^* | YBL011W | YKR067W | YBR042C | YDR018C | YDL052C | YOR245C | YPR140W | YPR139C | YOR298W |
| *Saccharomyces paradoxus^2^* | Spar_2.124 | Spar_11.357 | Spar_2.184 | Spar_4.300 | Spar_4.229 | Spar_15.451 | Spar_16.507 | Spar_16.506 | Spar_15.506 |
| *Saccharomyces mikatae^2^* | Smik_2.109 | Smik_11.329 | Smik_2.160 | Smik_4.252 | Smik_4.187 | Smik_15.429 | Smik_16.387 | Smik_16.386 | Smik_15.479 |
| *Saccharomyces kudriavzevii^2^* | Skud_2.100 | Skud_11.303 | Skud_2.154 | Skud_4.270 | Skud_4.203 | Skud_15.412 | Skud_16.434 | Skud_16.433 | Skud_15.465 |
| *Saccharomyces arboricolus^2^* |  | EJS42831 |  |  | EJS44387 | EJS41575 | EJS41281 | EJS41280 |  |
| *Saccharomyces bayanus^2^* | Sbay_2.114 | Sbay_11.302 | Sbay_4.274 | Sbay_2.168 | Sbay_4.198 | Sbay_8.295 | Sbay_16.468 | Sbay_16.467 | Sbay_8.347 |
| *Kazachstania africana^3^* | XP_003959018 | XP_003958593 | XP_003956191 |  | XP_003958479 | XP_003954956 | XP_003956089 | XP_003956088 | XP_003956927 |
| *Naumovozyma castellii^3^* | XP_ 003675136 | XP_003675893 | XP_003678186  XP_003673979 |  | XP_003672188 | XP_003674621 | XP_003677183 | XP_003677182 | XP_003674537 |
| *Candida glabrata^4^* | CAGL0E02849g | CAGL0K08162g | CAGL0I04620g |  | CAGL0I04070g | CAGL0J03674g | CAGL0D04972g | CAGL0D04950g | CAGL0A03806g |
| *Vanderwaltozyma polyspora^3^* | XP_001644985 | XP_001643508 | XP_001645319  XP_001647122 |  | XP_001643441  XP_001643328 | XP_001646192 | XP_001644119 | XP_001644118 | XP_001644271 |
| *Zygosaccharomyces rouxii^4^* | ZYRO0G09834g | ZYRO0B15708g | ZYRO0A03586g |  | ZYRO0B07326g | ZYRO0F07348g | ZYRO0B04312g | ZYRO0B04334g | ZYRO0C01034g |
| *Lachancea thermotolerans^4^* | KLTH0E07458g | KLTH0F17160g | KLTH0C08096g |  | KLTH0D13772g | KLTH0D11198g | KLTH0F14476g | KLTH0F14454g | KLTH0E04158g |
| *Kluyveromyces lactis^4^* | KLLA0B10538g | KLLA0F16104g | KLLA0B06820g |  | KLLA0E01233g | KLLA0F11165g | KLLA0B10978g | KLLA0B11000g | KLLA0F25058g |
| *Eremothecium gossypii^3^* | NP_986036 | NP_986967 | NP_983231 |  | NP_986139 | NP_983542 | NP_986920 | NP_986921 | NP_986071 |
| *Ogataea polymorpha^3^* | ESW99553 | ESW98880 | ESW97278 |  | ESW97514 | ESW99385 | ESX03647 |  |  |
| *Debaryomyces hansenii^4^* | DEHA2A08624g | DEHA2E07040g | DEHA2F15664g |  | DEHA2G01496g | DEHA0C13101g | DEHA0B02024g | DEHA0D17963g | DEHA0D18832g |
| *Millerozyma farinosa^4^* | PISO0F06935g  PISO0E05614g |  | PISO0I07774g  PISO0J09601g |  | PISO0L01397g  PISO0K01396g | PISO0H12899g  PISO0G12898g | PISO0M23574g  PISO0N23839g | PISO0N22607g  PISO0M22364g | PISO0B09131g  PISO0A09064g |
| *Meyerozyma guillermondii^3^* | EDK39427 | EDK41440 | EDK40103 |  | EDK40560 | EDK39486 | EDK38432 | EDK36520 | EDK36552 |
| *Candida albicans^5^* | XP_710056 | XP_715356 | XP_714065  XP_714343 |  | XP_720080 | XP_712742 | XP_714267 | XP_713693 | XP_721724 |
| *Komagataella pastoris^3^* | XP_002492283 | XP_002489418 | XP_002490189 |  | XP_002489498 | XP_002492428 | XP_002493075 | XP_002491337 | XP_002494158 |
| *Geotrichum candidum^6^* | GECA03s02265g |  | GECA13s01935g |  | GECA04s00340g  GECA01s06258g | GECA18s00813g | GECA09s04069g | GECA02s07787g |  |
| *Yarrowia lipolytica^4^* | YALI0C00209g |  | YALI0F02937g |  | YALI0E18964g | YALI0E32769g | YALI0C14036g | YALI0C14014g |  |
| *Neurospora crassa^3^* | XP_958638 |  | XP_958192 |  | XP_956681 | XP_965438 | XP_958270 | XP_958281 |  |
| *Aspergillus fumigatus^3^* | XP_751693 |  | XP_754044 |  | XP_755194 | XP_749138 | XP_755729 | XP_755755 |  |
| *Schizosaccharomyces pombe^3^* | NP_596450 |  | NP_595192 |  | NP_594605 | XP_001713160 |  | NP_593657 |  |
| *Cryptococcus neoformans^3^* | XP_569487 |  | XP_568878 |  | XP_567944  XP_570434 | XP_571235 | XP_777272 | XP_773987 |  |
